# Compressive strength prediction of coconut fiber reinforced concrete using PSO optimized explainable machine learning

**DOI:** 10.1038/s41598-026-56658-4

**Published:** 2026-06-05

**Authors:** Ahmed A. Alawi Al-Naghi, Tariq Ali, Inamullah Inam, Muhammad Sarmad Mahmood, Muhammad Zeeshan Qureshi, Khaled Mohamed Elhadi, Abdelkader Mabrouk, Ali Ajwad

**Affiliations:** 1 Civil Engineering Department, University of Ha’il, Ha’il, 55476 Saudi Arabia; 2 Department of Civil Engineering, Swedish College of Engineering and Technology, Wah, 47080 Pakistan; 3 Department of Civil Engineering, Engineering Faculty, Laghman University, Mehtarlam, Afghanistan; 4 Department of Civil Engineering, University of Engineering and Technology, Taxila, Pakistan; 5 Department of Civil Engineering, College of Engineering, King Khalid University, PO Box 394, Abha, 61411 Kingdom of Saudi Arabia; 6 Center for Engineering and Technology Innovations, King Khalid University, Abha, 61421 Saudi Arabia; 7 Civil Engineering Department, College of Engineering, Northern Border University, Arar, 73222 Saudi Arabia; 8 Department of Civil Engineering, University of Salerno, Fisciano, 84084 Italy

**Keywords:** Coconut Fiber Reinforced Concrete, Compressive Strength Prediction, Machine Learning, Particle Swarm Optimization, SHAP Explainability, Engineering, Materials science, Mathematics and computing

## Abstract

The use of natural fibers in concrete is a sustainable strategy to improve ductility and crack resistance; however, coconut fiber can introduce nonlinear interactions among mix constituents that complicate compressive strength prediction. This study proposes an optimized and explainable machine learning framework for compressive strength prediction of coconut fiber reinforced concrete (CFRC). Six regression algorithms, support vector machine (SVM), k-nearest neighbors (KNN), random forest (RF), light gradient boosting (LGB), extreme gradient boosting (XGB), and artificial neural network (ANN) were optimized using particle swarm optimization (PSO) on 586 experimental samples with eight inputs: cement, fine aggregate (FA), natural coarse aggregate (NCA), recycled coarse aggregate (RCA), water, fiber content, fiber length, and age. A nested grouped cross-validation scheme (outer 5-fold, inner 3-fold) was adopted for robust model selection, followed by evaluation on a locked 30% test set. The best model, XGB-PSO, achieved mean cross-validation performance of R^2^ = 0.963 ± 0.013, RMSE = 3.018 ± 0.646 MPa, MAPE = 7.744 ± 1.008% and demonstrated strong generalization on the locked test set (R^2^ = 0.953, RMSE = 3.71 MPa, MAPE = 8.875%). Model explainability using shapley additive explanations (SHAP), partial dependence plots (PDP), accumulated local effects (ALE), and individual conditional expectation (ICE) consistently identified age and RCA as dominant predictors, supporting data driven CFRC mix design and optimization.

**Supplementary Information:**

The online version contains supplementary material available at 10.1038/s41598-026-56658-4.

## Introduction

Concrete is the world’s most widely used construction material, but it faces significant sustainability and durability challenges. The production of cement, the key binding component in concrete is highly carbon intensive, making concrete responsible for roughly 8–10% of global CO_2_ emissions^1–3^. This substantial carbon footprint raises concerns about climate impact and resource consumption in the construction sector. At the same time, conventional concrete can suffer from durability issues like cracking, shrinkage, and deterioration under harsh environments, which shorten the service life of structures and necessitate frequent repairs^4^. These limitations drive an urgent need for more sustainable and longer-lasting concrete solutions worldwide.

In response to these challenges, the construction industry is innovating towards greener and more durable concrete. One major approach is the use of supplementary cementitious materials (SCMs) such as fly ash, slag, and silica fume to partially replace Portland cement, thereby cutting the carbon footprint of concrete. Emerging technologies like alkali-activated binders (geopolymers), carbon capture utilization during cement production, and the use of recycled aggregates have shown the potential to reduce concrete’s CO_2_ emissions by roughly 30–50%^5^. In parallel, advanced mix design strategies and chemical admixtures are being employed to enhance durability, for instance, optimizing particle packing or incorporating fibers to control cracking. Such measures can improve concrete durability by 20–25% and reduce lifecycle costs through diminished maintenance needs^5^.

Fiber-reinforced concrete (FRC) has proven to be a powerful strategy for improving the mechanical behaviour and longevity of concrete^6^. In FRC, discrete short fibers (commonly steel, polymer, glass, or natural fibers) are dispersed throughout the cement matrix, bridging micro-cracks and providing post-cracking ductility^7^. Even at low volume fractions, fibers significantly mitigate crack propagation and enhance the material’s resistance to tensile stress, impact, and abrasion. By reducing crack formation and widening, fiber additions lead to greater structural integrity and resilience. As a result, FRC elements exhibit improved durability and extended service life compared to plain concrete, with fewer repairs required over their lifespan^4,8,9^. This improvement in performance has made fiber reinforcement a key component of modern high-performance and sustainable concrete designs aimed at greater durability.

Among the various fiber options, natural fibers have attracted considerable interest for their sustainability and cost advantages. Coconut fiber, in particular, stands out as an abundantly available natural fiber obtained from coconut husks, an agricultural waste product in tropical regions that is often disposed of in landfills^10^. Utilizing coconut coir as concrete reinforcement provides productive use for this waste while offering a low-cost alternative to synthetic or steel fibers. Natural fibers like coir are lightweight and can reduce the density of concrete, and their inclusion has been shown to limit crack propagation, thereby improving the toughness of the composite^11–13^. Moreover, being plant-based and locally available, coir fibers are renewable, and their production requires minimal energy; unlike steel fibers, they do not significantly increase the embodied carbon of the concrete. For example, adding just 1% steel fier can increase concrete material cost by over 90% and its arbon footprint by about 50%, whereascoconut fibers incorporate negligible additional cost and environmental impact^14,15^. These characteristics make coconut fiber a highly attractive reinforcement for eco-friendly concrete applications.

Coconut fiber also exhibits excellent mechanical properties that contribute to concrete performance. It has high tensile strength and remarkable ductility reportedly the highest tensile strength among common natural fibers allowing coir to sustain several times more deformation than other fibers before failure^16^. This enables coconut fibers to act as effective crack-bridging reinforcements, improving the ductility and post-crack load-bearing capacity of the concrete. Recent studies have documented notable enhancements in the mechanical performance of concrete with coconut fiber inclusion. For instance, adding a small proportion of coir (on the order of 0.5–1.5% b volume) was found to increase the 28-day compressive strength of concrete by roughly 15–25% cmpared to a plain mix^10,17,18^, while also significantly boosting flexural strength and impact resistance. However, excessively high fiber content (e.g. above ~ 4% by volume) an diminish workability and even reduce compressive strength due to fiber agglomeration and voids, indicating an optimal fiber dosage for best results^19^. At appropriate fiber levels, the overall toughness and energy absorption capacity of the concrete are substantially improved. In one study, coconut fiber reinforced concrete achieved about a 13% higher compressve strength than the control mix (at 28 days) and showed lower permeability and chloride penetration, demonstrating superior durability under aggressive environmental conditions^20^. These findings underscore that coconut fibers can effectively enhance both the strength and longevity of concrete when used judiciously.

In recent years, ML techniques have emerged as powerful tools for predicting the properties of concrete, helping to streamline mix design and minimize reliance on conventional trial-and-error procedures^21–25^. Traditionally, determining the compressive strength of a concrete mix requires extensive laboratory testing involving multiple casting and curing cycles, which is both labor intensive and time-consuming^26–29^. This challenge becomes more pronounced for non-conventional materials such as CFRC, where the interaction between fiber dosage, length, and mix constituents introduces highly nonlinear and coupled effects that conventional empirical or mechanistic mix-design models struggle to represent accurately. ML offers a practical alternative for pre-mix design screening and lab data augmentation, enabling rapid strength estimation from mix parameters without exhaustive physical testing^24^. By training regression models on prior experimental datasets, ML can accurately predict compressive strength based on mix composition and curing age, providing a foundation for more efficient proportioning and quality control. A variety of algorithms such as support vector machines, artificial neural networks, decision trees, and ensemble methods have been successfully applied to concrete strength prediction^30^. These models often explain over 90% of the strength varability^31^, significantly reducing testing time and cost while improving optimization of materials for target performance. Hence, in the case of coconut fiber concretes, ML-based models are particularly advantageous as they capture the complex, nonlinear interactions between fiber geometry, dosage, and binder characteristics offering more reliable predictions than traditional mechanistic or empirical approaches^5^.

However, despite rapid progress in ML for concrete property prediction, dedicated models for natural fiber–reinforced concretes such as CFRC remain limited, and existing studies often rely on modest datasets and provide restricted interpretability for mix design^31^. The mechanical response of CFRC is governed by nonlinear interactions among fiber dosage/geometry and mixture constituents, which conventional empirical approaches and generic ML pipelines may not capture reliably^10^. To address this gap and support sustainability-driven mix optimization (reduced trial batching, material waste, and reliance on carbon-intensive binders), this study develops six PSO optimized ML learners (SVM, KNN, RF, LGB, XGB, and ANN) and integrates explainability using SHAP with complementary PDP, ALE, and ICE analyses to clarify predictor importance and nonlinear effects. Using a compiled dataset of 586 CFRC samples, the study aims to achieve four objectives i.e., (i) compare PSO-tuned models under a nested grouped cross-validation protocol, (ii) evaluate generalization on a locked test set, and (iii) provide interpretable insights for practical CFRC mix proportioning, (iv) assess relative accuracy and stability using Taylor diagrams and error metrics for final model ranking.

## Literature review and research significance

Recent studies have successfully applied ML to predict the CS of fiber-reinforced cement composites^6,21–24,32,33^. For example, Kashyap et al.^34^ compiled 103 data points of jute-fiber reinforced concrete and used soft computing models (ANFIS, ANN, Random Forest, Random Tree) to predict CS. They used only three input features (fiber aspect ratio, fiber volume percentage, curing days) and found Random Forest to be the most accurate (correlation coefficient CC ≈ 0.987 on training data). Ahmad et al.^35^ studied sisal-fiber reinforced concrete using 68 mixes (data from literature) with six input variables (cement, sand, coarse aggregate, water/cement ratio, fiber content, and curing time). They compared SVM, Gaussian Process, ANN, linear and nonlinear regression, and found that an ANN gave the best performance (highest R^2^ and lowest RMSE). In another work, Moraes et al.^36^ developed ANN models (with Extreme Learning) to predict strengths of mortar reinforced with babassu coconut fiber. They generated 51 lab samples and used six inputs (cement, sand, w/c, maximum fiber length, fiber %, slump); both tensile and compressive strength models were “promising” for prediction. Similarly, Kiran et al.^37^ examined coconut-fiber reinforced paver blocks (with construction/demolition waste replacing fine aggregate) and applied RSM, SVM, Gradient Boosting, ANN, and RF to predict compressive strength. In their 36 experimental mixes, five inputs were used (cement, natural fine aggregate, recycled aggregate, CDW content, coconut fiber%) and they reported that ensemble models (LGB and ANN) outperformed SVM and RF. In fact, RF had the highest accuracy (R^2^ ≈ 0.935) for CS prediction.

Furthermore, recent ML studies on cementitious materials provide useful methodological evidence for model selection and interpretability. For example, Jueyendah et al.^38^ used SVM with different kernels to predict mortar mechanical properties and reported that the RBF kernel performed best, with improved accuracy when porosity was included as an input; for SVM–RBF, RMSE values of 0.2909 (R^2^ = 0.9970) and 1.2969 (R^2^ = 0.9987) were reported for flexural and compressive strength prediction, respectively. Moreover, Jueyendah et al.^39^ compared a broad range of linear and non-linear ML algorithms for mortar strength estimation and found that a systematically configured neural network achieved excellent testing performance (R^2^ = 0.9946, RMSE = 1.5032, MAE = 1.2545) and used SHAP to show that curing age and nano silica/cement ratio positively influence strength, while porosity has a dominant negative influence.

All these studies demonstrate that ML can effectively capture complex mix-design effects on strength^40,41^, but they share common limitations. In particular, previous studies have generally employed basic interpretability methods, such as sensitivity analyses, and have typically been conducted with relatively modest dataset sizes. For instance, while a notable study on basalt-fiber reinforced concrete utilized 309 data points^42^, many machine learning applications involving natural fiber concretes have been based on datasets of around 50–100 samples^34–36^. Moreover, most prior research has primarily focused on fiber volume or percentage as a primary input parameter, with relatively few explicitly incorporating fiber length as an influential variable; a notable exception is the study by Moraes et al.^36^, which included fiber length in their predictive modeling. Overall, existing research provides valuable insights into the prediction of compressive strength for concrete reinforced with fibers like jute, sisal, and coconut. However, opportunities remain to enhance the comprehensiveness and interpretability of these models through the use of larger datasets, inclusion of additional influential parameters like fiber length, and application of advanced explainability tools.

### Research significance

This study provides a robust and practically useful contribution to the emerging domain of CFRC by addressing key limitations in existing ML-based strength prediction research. Most prior CFRC-focused studies remain constrained by moderate dataset sizes and incomplete representation of fiber geometry, while interpretability is often limited to basic sensitivity analysis, as reflected in the notable study by Kashyap et al.^31^ employing a relatively larger dataset of 192 samples, most existing research still relies on moderately sized datasets, typically in the range of 50–103 samples. Additionally, although fiber content is frequently included as an influential parameter, fiber geometry particularly fiber length has often been less commonly addressed, with only a few notable exceptions such as the mortar study by Moraes et al.^36^. Furthermore, while traditional sensitivity analyses have provided valuable insights, the use of advanced interpretability techniques such as SHAP, PDP, ALE or ICE plots remains relatively underexplored. Collectively, these gaps indicate a clear need for robust and interpretable predictive models for coconut fiber-reinforced concrete, using larger datasets, comprehensive parameter inclusion (notably fiber length), and advanced explainability methods. In response, this study advances the state of knowledge by assembling a comparatively large dataset of 586 CFRC samples to enhance generalization, explicitly incorporating fiber length alongside fiber content to capture a critical geometric variable, and integrating PSO across multiple algorithms (SVM, KNN, RF, LGB, XGB, ANN) with explainability tools (SHAP supported by PDP, ALE, and ICE) to reveal dominant predictors and nonlinear effects. Beyond improving predictive accuracy, this integrated and interpretable framework strengthens scientific rigor and practical relevance by enabling transparent, data-driven guidance for CFRC mix design and performance optimization, thereby extending current practice beyond standard tuning or sensitivity analysis.

## Research methodology

### Data characteristics

This study utilizes a comprehensive dataset compiled from 30 research articles, which collectively provide a total of 586 experimental data points related to CFRC. The dataset sources are provided in Table 1 and the complete dataset is provided in the supplementary file. Since the data were collected from different literature sources, the samples may differ in terms of laboratory conditions, raw material properties, mix design protocols, specimen preparation methods, and curing regimes. Therefore, the publication source was treated as a grouping variable during data splitting to ensure that samples from the same source were strictly confined to either the training/development subset or the locked test subset.

**Table 1:** Data sources and counts for the compiled CFRC dataset.

Sr. #	Refs.	X	Sr. #	Refs.	X	Sr. #	Refs.	X
1.	^43^	05	**2.**	^44^	12	**3.**	^45^	21
4.	^18^	10	**5.**	^46^	25	**6.**	^47^	168
7.	^48^	12	**8.**	^49^	11	**9.**	^50^	08
10.	^51^	04	**11.**	^52^	02	**12.**	^53^	12
13.	^54^	30	**14.**	^55^	18	**15.**	^56^	04
16.	^57^	15	**17.**	^58^	28	**18.**	^59^	12
19.	^60^	08	**20.**	^61^	20	**21.**	^62^	18
22.	^63^	15	**23.**	^19^	20	**24.**	^64^	17
25.	^65^	15	**26.**	^66^	18	**27.**	^67^	10
28.	^68^	08	**29.**	^69^	32	**30.**	^70^	08
Total								**586**

X: Data Counts.

Inclusion criteria required that mixes reported compressive strength together with all eight input features. Mixes were excluded if essential parameters such as fiber dosage or curing age were missing. To avoid duplication, repeated datasets across studies were cross-checked and retained only once. After the source-based train/test split, outlier screening was performed only on the training/development subset by inspecting extreme values of fiber dosage, fiber length, and compressive strength. Potentially implausible values were verified against the original sources before exclusion or correction. However, a small number of borderline outliers were retained intentionally to preserve the natural variability of experimental data and to ensure the machine learning models remained robust across a realistic range of mix proportions and mechanical performance.

The primary objective is to predict the CS (MPa) of concrete based on key mix design parameters. Each data point includes eight input features such as cement content (kg/m^3^), coconut fiber length (mm), coconut fiber percentage (%), natural coarse aggregate (NCA) (kg/m^3^), recycled coarse aggregate (%), fine aggregate (FA) (kg/m^3^), age (Days) and one output i.e., compressive strength (CS) (MPa).

To understand the distribution and quality of the dataset without introducing information leakage, descriptive statistical analysis was performed only on the training/development subset after the source-based split. Table 2 provides the data dictionary including unit, range, mean, median, mode, standard deviation, variance, skewness, and kurtosis for each variable. The locked test subset was excluded from this exploratory analysis and was retained only for final model evaluation.

**Table 2:** Descriptive statistics of the training/development dataset used for model development.

Parameters	Unit	Range	Mean	Median	Mode	Std	Variance	Skewness	Kurtosis
Cement	kg/m^3^	255.00–550.00	406.82	400	320	78.59	6175.93	0.09	−1.29
Fiber Length	mm	0.00–200.00	38.25	35	50	33.99	1155.17	3.17	12.72
Fiber Content	%	0.00–12.00	1.95	1.5	1	1.82	3.32	1.69	4.78
NCA	kg/m^3^	0.00–1400.00	942.47	1075	1171.2	358.93	128830.5	−1.13	0.29
RCA	%	0.00–10.00	1.24	0	0	2.72	7.39	2.42	4.78
FA	kg/m^3^	560.00–1052.00	752.37	710.4	710.4	121.04	14649.78	0.62	−0.16
Water	kg/m^3^	160.00–300.00	196.49	180	175	34.17	1167.92	1.56	1.85
Age	Days	3.00–546.00	29.19	28	28	54.95	3019.5	6.56	50.28
CS	MPa	10.00–86.30	31.77	27.25	30	15.99	255.73	1.48	1.76

The mean represents the average value of each feature, while the median indicates the central tendency, which is particularly useful in the presence of skewed data. The mode reflects the most frequently occurring value. For example, the mean compressive strength in the training/development subset is 31.77 MPa, while the median is slightly lower at 27.25 MPa, indicating a slight right-skew. The range values provide a clear sense of the spread of each parameter, for example, compressive strength varies from 10.0 to 86.3 MPa. The standard deviation and variance further quantify the dispersion of the data. For instance, compressive strength has a standard deviation of 15.99 MPa, indicating notable variability in mechanical performance due to mix design and curing conditions.

Higher-order statistics such as skewness and kurtosis offer deeper insight into data distribution. A skewness value greater than 1, such as 6.56 for Age and 1.69 for fiber content, indicates a right-skewed distribution. Likewise, high kurtosis values, such as 50.28 for Age, suggest the presence of outliers or heavy tails in the training/development data, which is expected due to variations in experimental curing periods across different studies.

**Fig. 1:**
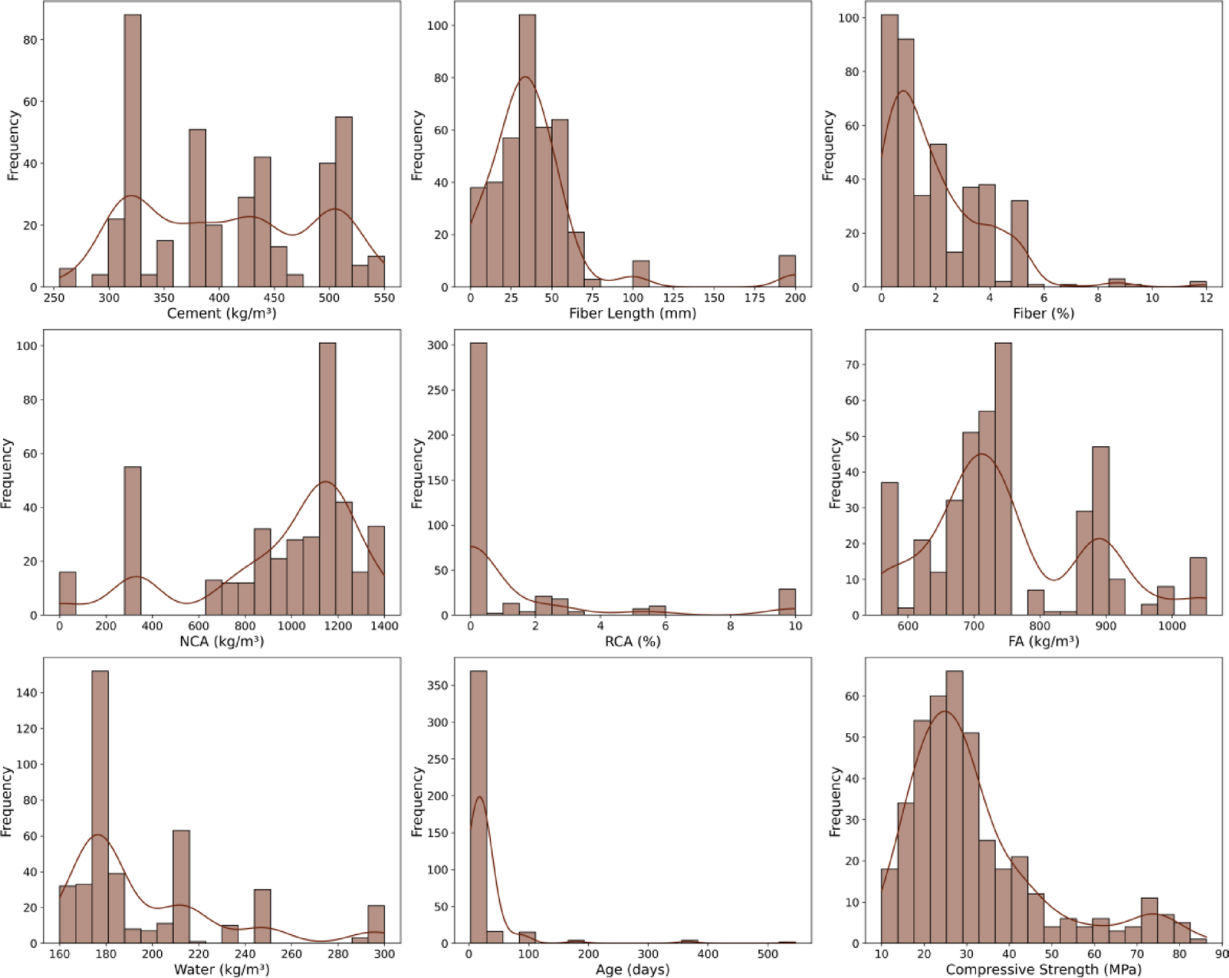
Frequency distribution of input and output variables in the training/development dataset.

To visualize the spread and distribution of the training/development dataset, histograms for all variables are shown in Fig. 1. Each subplot depicts the frequency distribution and probability density curve of each feature. As evident from the histograms, cement content, water, NCA, and FA show multi-modal distributions, reflecting variability in mix design strategies across different literature sources. Fiber content and RCA are strongly right skewed, with most data concentrated at lower values, indicating that most mixes contain low to moderate levels of coconut fiber and RCA. Fiber length is mainly concentrated between approximately 20 and 60 mm, with a few longer fibers extending up to 200 mm. Age (days) is sharply skewed with a strong concentration around 28 days, which is the standard curing age for strength testing, while a few samples extend to longer curing durations.

### Multicollinearity assessment

**Fig. 2:**
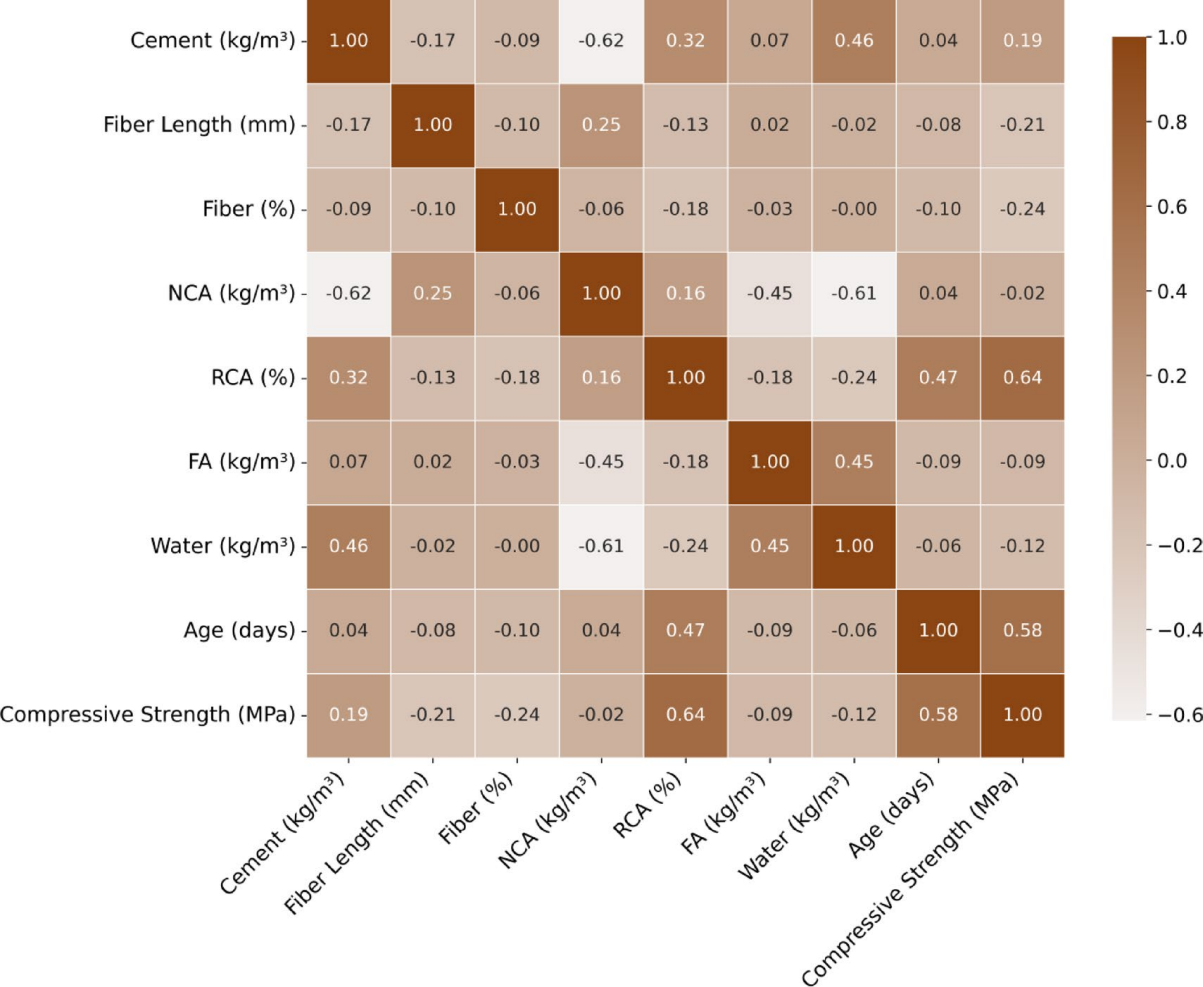
Pearson correlation heatmap for multicollinearity assessment based on the training/development dataset.

Multicollinearity occurs when two or more input features are highly linearly correlated, which can distort model interpretations, inflate error terms, and lead to unstable predictions, especially in regression-based models. To evaluate this without information leakage, a Pearson correlation coefficient matrix was generated using only the training/development subset and is presented as a heatmap in Fig. 2.

A commonly used threshold for potential multicollinearity is an absolute Pearson correlation of $\:\mid\:r\mid\:>0.70$, beyond which variables are considered highly correlated and potentially redundant^71,72^. In this study, the training-set correlation matrix reveals that all pairwise correlations between input features are below this threshold, suggesting an absence of severe multicollinearity. For instance, moderate correlations are observed between NCA and cement (− 0.62), NCA and water (− 0.61), water and cement (0.46), and FA and water (0.45), but all remain below the critical 0.70 mark. The strongest correlations with compressive strength are observed for RCA (0.64) and age (0.58), indicating their strong relevance to strength prediction without creating problematic collinearity among the input variables.

Given this outcome, all eight input variables were retained for model training without the need for dimensionality reduction or feature elimination, as no pairs of input features exhibit problematic collinearity. This ensures that the machine learning algorithms can fully leverage the underlying relationships between mix design parameters and compressive strength. The training-set-based statistical and graphical analysis confirms the dataset’s richness and heterogeneity, making it suitable for developing robust and generalizable machine learning models for compressive strength prediction.

### Feature engineering and normalization

In this study, no additional feature transformations or engineered variables were introduced, as the original eight input parameters, cement, fiber length, fiber percentage, NCA, RCA, FA, water, and age are all physically meaningful and directly relevant to the prediction of compressive strength. Since all features are already numerical in nature, no encoding or conversion of categorical variables was necessary.

Furthermore, the input features were normalized using Z-score standardization, also known as StandardScaler. This method transforms each feature so that it has a mean of zero and a standard deviation of one. This ensures that all input features are on a comparable scale, promoting model convergence and stability during optimization. Each feature $\:{x}_{i}$ was scaled according to Eq. (1).

1 $$\:{x}_{i}^{{\prime\:}}=\frac{{x}_{i}-{u}_{i}}{{\sigma\:}_{i}}$$

Where $\:{u}_{i}$ and $\:{\sigma\:}_{i}$ represent the mean and standard deviation of the training data only. The scaler was fit exclusively on the training subset and subsequently applied to the validation and test sets to avoid any information leakage. This normalization was applied only to models that rely on gradient descent or distance metrics namely SVM, KNN, and ANN. Tree-based ensemble models (RF, LGB, and XGB) inherently handle unscaled data, and therefore no normalization was applied to them.

### Data splitting and cross-validation

A nested grouped cross-validation (CV) protocol was adopted to ensure unbiased performance estimation and to prevent information leakage across data partitions. The dataset comprised samples collected from multiple experimental studies, each contributing several observations under related laboratory conditions. To account for this structure, a grouping variable representing the publication source was used throughout all stages of data splitting. This ensured that all observations originating from the same study were confined to a single subset, thereby preserving independence between training and evaluation data. Accordingly, all exploratory analyses, including descriptive statistics, histogram visualization, correlation analysis, and outlier screening, were performed only after this source-based split and using the training/development subset, while the locked test subset remained untouched until final evaluation.

Initially, a locked test subset representing 30% of the total data was separated using a ‘Group Shuffle Split’ procedure with a fixed random seed of 42 to guarantee reproducibility. This subset remained completely unseen during both training and hyperparameter tuning and was reserved exclusively for the final model validation. The remaining 70% of the grouped data served as the basis for a two-level nested CV design, comprising an outer five-fold loop for generalization assessment and an inner three-fold loop for hyperparameter optimization. Both loops employed Group K Fold partitioning to maintain consistent grouping boundaries across folds.

Within each outer-CV iteration, the inner loop guided the search for optimal hyperparameters through PSO, which minimized the mean root-mean-square error across the inner folds. The model was subsequently retrained on the full outer-training portion using the tuned parameters and evaluated on the corresponding outer-test fold, producing fold-wise estimates of performance metrics such as R^2^, RMSE, RRMSE, NRMSE, MAE, MAPE, and Median AE. The outcomes from all five outer folds were aggregated to obtain the mean ± standard deviation and 95% confidence intervals (CI) reported in the results.

Following nested evaluation, the best-performing hyperparameters were averaged across the outer folds and used to train the final model on the entire 70% training subset. This trained model was then applied to the locked 30% test set to obtain the definitive performance values reported in this study. The described nested grouped CV framework provides a statistically robust and leakage-free protocol that reflects realistic model generalization to unseen experimental studies.

### Model development and optimization framework

The hyperparameter tuning process for each predictive model was conducted using PSO integrated within the inner loop of the nested cross-validation framework. PSO is a population-based stochastic optimization algorithm inspired by the collective foraging and movement behavior of birds. It iteratively adjusts the population of candidate solutions, termed particles, to minimize a predefined objective function^73,74^. In this study, the root mean squared error (RMSE) across the inner validation folds. During optimization, each particle updates its position in the multidimensional search space based on both its personal best-known position and the global best identified by the swarm, thereby achieving an effective balance between exploration and exploitation.

To ensure methodological consistency in comparative evaluation, the PSO configuration was standardized across all models. The swarm comprised 20 particles, and optimization proceeded for up to 25 iterations. The inertia weight (ω), regulating the balance between global and local exploration, was set to 0.7, while both the cognitive (φ_p_) and social (φ_g_) acceleration coefficients were fixed at 1.5, controlling the influence of individual and collective learning experiences, respectively. Parameter boundaries were customized according to each model’s hyperparameter domain (e.g., learning rate, tree depth, regularization term, or number of neurons), as detailed in the corresponding Table 4. During each PSO iteration, candidate configurations were trained on the inner training folds and validated on the inner validation folds using grouped data splits. The objective function computed the average RMSE across inner folds, and the parameter set yielding the lowest value was identified as optimal. This optimal configuration was subsequently retrained on the full outer-training subset for unbiased evaluation. The integration of PSO within the nested grouped cross-validation framework ensured a leak-free and statistically strong hyperparameter optimization process, maintaining independence from the locked test subset and reinforcing methodological reliability.

To ensure robust benchmarking for the nonlinear behavior of CFRC, six complementary regression learners were selected, representing distinct modelling families: kernel-based learning (SVM), instance-based local regression (KNN), bagging-based ensembles (RF), boosting-based ensembles (LGB and XGB), and neural networks (feed-forward ANN). This selection provides both strong nonlinear function approximation and diversity in inductive bias as tree ensembles and boosting models are effective for capturing feature interactions and nonlinearity, with XGB widely reported as a high-performing scalable boosting framework, while LGB is designed for efficient gradient boosting decision trees^75,76^. SVM and KNN were included as well-established baselines for nonlinear regression and local interpolation, respectively^35,40^, and ANN was included to capture complex nonlinear mappings^77–79^. Hyperparameters were tuned using PSO due to its effectiveness for continuous, non-convex search spaces with minimal assumptions^80^.

#### K-nearest neighbors

KNN is a non-parametric algorithm that predicts outputs by averaging the values of the k-nearest training samples in the feature space. It is simple to implement and interpret but can be sensitive to the choice of k and feature scaling^81^.

#### Support vector machine

SVM is a powerful supervised learning algorithm that constructs a hyperplane to separate data with maximum margin. For regression tasks, it fits the best function within a defined error margin^82^. Hyperparameters such as the penalty term C, kernel type, and gamma significantly affect performance.

#### Random forest

RF is an ensemble method that builds multiple decision trees and averages their predictions to reduce overfitting^83^. It is robust, handles non-linearity well, and performs well with limited hyperparameter tuning^84^. Parameters such as number of trees (estimators) and tree depth (max depth) were fine-tuned using PSO.

#### Extreme gradient boosting

XGB is a highly efficient and scalable implementation of gradient boosting that adds trees sequentially to minimize prediction error^85^. Due to its sensitivity to hyperparameters (e.g., learning rate, estimators, max depth, subsample)^86^, PSO was used to optimize these parameters for enhanced performance and reduced overfitting.

#### Light gradient boosting machine

LGB is another gradient boosting algorithm known for its fast-training speed and low memory usage. It uses histogram-based decision tree learning^87,88^. Like XGB, its key parameters such as learning rate, number of leaves, and max depth were optimized using PSO to maximize predictive accuracy.

#### Artificial neural network

ANNs are bio-inspired models that are well-suited for capturing complex, non-linear relationships in data^77–79^. The architecture used in this study included an input layer (8 features), hidden layers (optimized neuron counts), and one output neuron for compressive strength prediction. The Adam optimizer was used for weight updates during training. Simultaneously, PSO was applied to tune the network’s hyperparameters (e.g., number of hidden layers, learning rate, and batch size), allowing the ANN to reach optimal architecture and convergence conditions.

### Model evaluation

To assess the performance of the developed machine learning models, several widely used statistical metrics were employed. These metrics quantify the accuracy of predicted compressive strength values against experimental data. The following seven primary evaluation criteria were used:

1. **Coefficient of Determination (R**^**2**^**)**: Indicates the proportion of variance in the dependent variable that is predictable from the independent variables and is defined in Eq. (2).

2 $$\:{R}^{2}=\:1-\frac{{{\Sigma\:}}_{i=1}^{n}{\left({x}_{i}-{\widehat{x}}_{i}\right)}^{2}}{{{\Sigma\:}}_{i=1}^{n}{\left({x}_{i}-\stackrel{-}{x}\right)}^{2}}$$

2. **Root Mean Squared Error (RMSE)**: Measures the average magnitude of the prediction error. It penalizes larger errors more than MAE and is defined in Eq. (3).

3 $$\:RMSE=\sqrt{\frac{1}{n}{\sum\:}_{i=1}^{n}{\left({x}_{i}-{\widehat{x}}_{i}\right)}^{2}}$$

3. **Normalized Root Mean Squared Error (NRMSE)**: Normalizes RMSE relative to the mean of observed values, allowing comparison across datasets. NRMSE was computed from Eq. (4).

4 $$\:NRMSE=\:\frac{RMSE}{\stackrel{-}{x}}$$

4. **Relative RMSE (RRMSE)**: Increasingly used in materials science and civil engineering, RRMSE expresses RMSE relative to the range of observed values and is defined in Eq. (5). This provides insight into model error relative to the observed spread in data.

5 $$\:RRMSE=\:\frac{RMSE}{{x}_{max}-{x}_{min}}\times\:100$$

5. **Mean Absolute Error (MAE)**: Represents the average of the absolute differences between predicted and actual values. MAE was computed using Eq. (6).

6 $$\:MAE=\:\frac{1}{n}{\sum\:}_{i=1}^{n}\left|{x}_{i}-{\widehat{x}}_{i}\right|$$

6. **Mean Absolute Percentage Error (MAPE)**: Expresses prediction error as a percentage of actual values, making it interpretable in relative terms and is defined in Eq. (7).

7 $$\:MAPE=\:\frac{100\mathrm{\%}}{n}{\sum\:}_{i=1}^{n}\left|\frac{{x}_{i}-{\widehat{x}}_{i}}{{x}_{i}}\right|$$

7. **Median Absolute Error (Med AE)**: Represents the median of absolute differences between predicted and actual values, offering robustness against outliers and is defined in Eq. (8).

8 $$\:Med\:AE=median\left(\left|{x}_{i}-\:{\widehat{x}}_{i}\right|\right)$$

## Results and discussion

### Cross-validation performance and hyperparameter optimization

This section addresses Objective 1, which compares the predictive performance of six PSO-optimized learners (SVM, KNN, RF, LGB, XGB, ANN) using nested cross-validation. The following subsections describe the model training setup, computational efficiency, and parameter tuning outcomes.

#### Nested cross-validation results

**Fig. 3:**
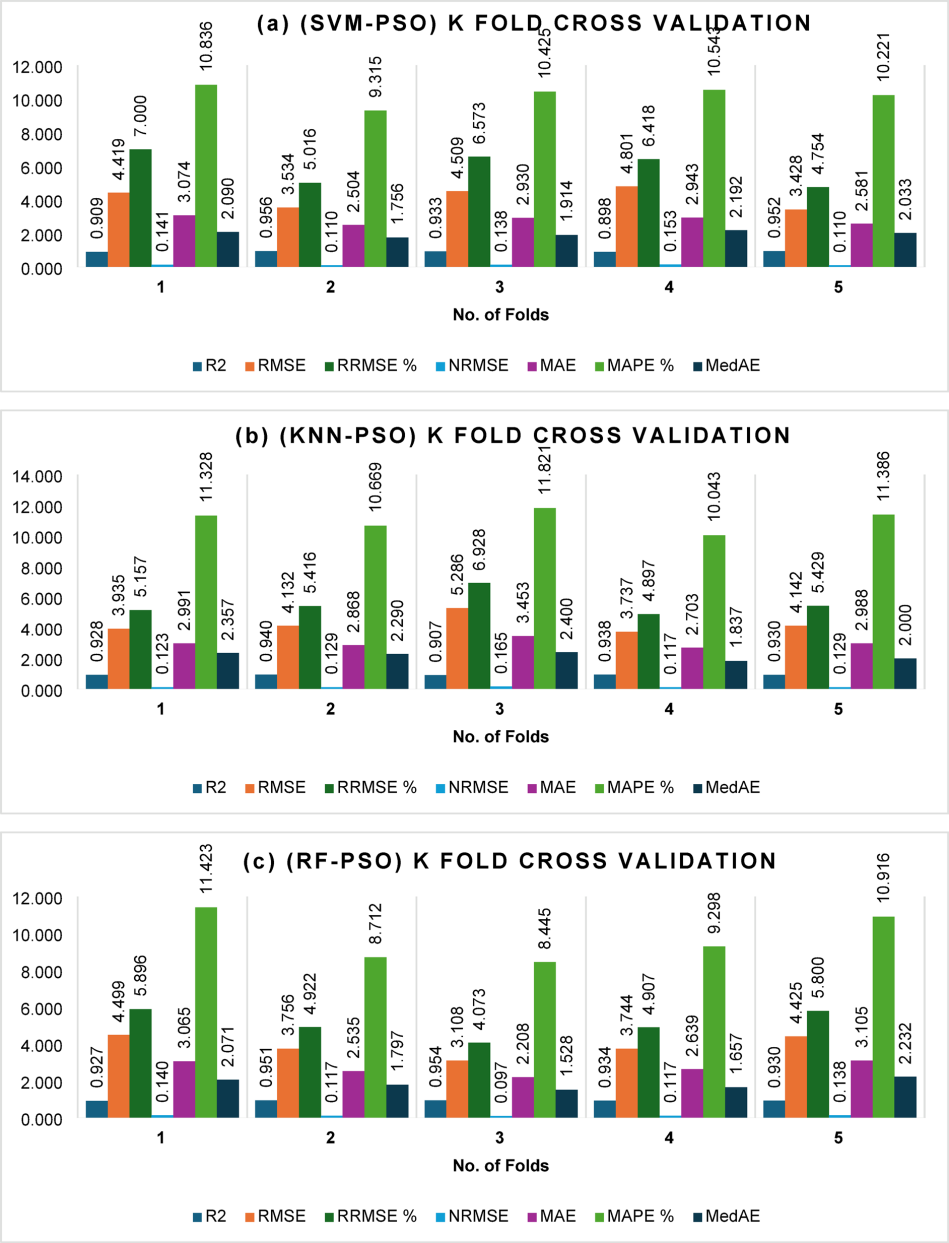

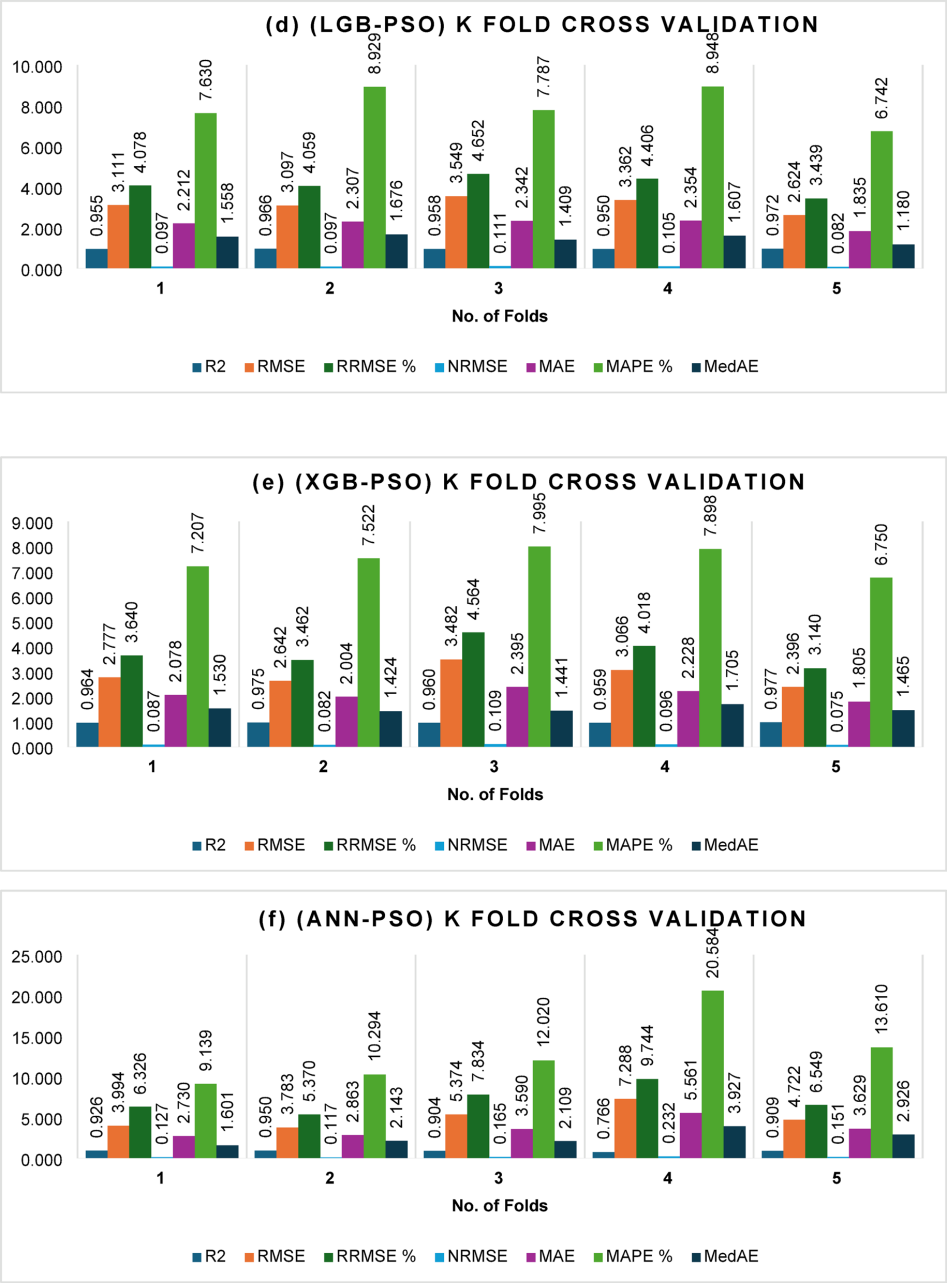
Performance evaluation of the PSO-optimized using 5-fold cross-validation for models (**a**) SVM-PSO, (**b**) KNN-PSO, (**c**) RF-PSO, (**d**) LGB-PSO, (**e**) XGB-PSO, (f) ANN-PSO.

Predictive performance was assessed under a five-fold nested, grouped cross-validation design (outer folds for performance estimation; inner folds for PSO tuning). Figure 3 (a-f) report per-fold metrics, and Table 3 summarizes Mean ± SD across the five outer folds.

XGB-PSO delivered the most accurate and stable predictions overall, achieving the highest mean R^2^ = 0.963 ± 0.013 with the lowest errors among all models **(**RMSE = 3.018 ± 0.646 MPa, RRMSE = 4.331 ± 0.942%, NRMSE = 0.095 ± 0.019, MAE = 2.151 ± 0.393 MPa, MAPE = 7.744 ± 1.008%, Med AE = 1.570 ± 0.348 MPa). The LGB-PSO model followed closely with R^2^ = 0.960 ± 0.009 and RMSE = 3.149 ± 0.348 MPa, demonstrating similarly high predictive accuracy. Among the remaining models, RF-PSO showed competitive performance with R^2^ of 0.938 ± 0.015 and RMSE of 3.929 ± 0.555 MPa. SVM-PSO (R^2^ = 0.930 ± 0.026) and KNN-PSO (R^2^ = 0.929 ± 0.013) delivered moderate accuracy, while their error metrics (MAPE > 11%) suggested sensitivity to feature scaling and hyperparameter variation. The ANN-PSO configuration was comparatively weaker (R^2^ = 0.891 ± 0.072, RMSE = 5.032 ± 1.409 MPa, MAPE = 13.129 ± 4.50%), reflecting higher fold-to-fold variability despite early stopping and PSO tuning.

Taken together, the boosting-based models (XGB-PSO > LGB-PSO > RF-PSO) demonstrated the most favorable balance of accuracy and robustness under grouped, nested validation. The associated 95% confidence intervals reported in Table 3 corroborate these findings, with XGB-PSO exhibiting the narrowest uncertainty bands among the top performers.

**Table 3:** Cross-validation performance of PSO optimized models (mean ± SD and 95% CI).

Models	Metrics	*R* ^2^	RMSE (MPa)	RRMSE* (%)	NRMSE*	MAE (MPa)	MAPE (%)	Med AE (MPa)
SVM-PSO	**Mean ± SD**	0.930 ± 0.026	4.138 ± 0.618	5.952 ± 1.001	0.130 ± 0.019	2.806 ± 0.249	10.268 ± 0.577	1.997 ± 0.168
	**95% CI**	0.022	0.541	0.878	0.017	0.218	0.506	0.148
	**LB**	0.907	3.597	5.074	0.113	2.588	9.762	1.849
	**UP**	0.952	4.679	6.830	0.147	3.024	10.774	2.145
KNN-PSO	**Mean ± SD**	0.929 ± 0.013	4.246 ± 0.604	5.565 ± 0.792	0.132 ± 0.019	3.001 ± 0.279	11.049 ± 0.697	2.177 ± 0.246
	**95% CI**	0.013	0.487	0.674	0.014	0.317	1.365	0.329
	**LB**	0.925	3.442	4.959	0.109	2.457	8.759	1.483
	**UP**	0.952	4.416	6.306	0.138	3.091	11.489	2.142
RF-PSO	**Mean ± SD**	0.938 ± 0.015	3.929 ± 0.555	5.633 ± 0.769	0.124 ± 0.016	2.774 ± 0.362	10.124 ± 1.557	1.813 ± 0.376
	**95% CI**	0.011	0.530	0.694	0.017	0.244	0.611	0.216
	**LB**	0.917	3.717	4.871	0.116	2.756	10.438	1.961
	**UP**	0.940	4.776	6.260	0.149	3.245	11.660	2.393
LGB-PSO	**Mean ± SD**	0.960 ± 0.009	3.149 ± 0.348	4.127 ± 0.456	0.098 ± 0.011	2.210 ± 0.217	8.007 ± 0.939	1.486 ± 0.197
	**95% CI**	0.008	0.305	0.400	0.010	0.190	0.823	0.173
	**LB**	0.953	2.843	3.727	0.089	2.020	7.184	1.313
	**UP**	0.968	3.454	4.527	0.108	2.400	8.830	1.659
XGB-PSO	**Mean ± SD**	0.963 ± 0.013	3.018 ± 0.646	4.331 ± 0.942	0.095 ± 0.019	2.151 ± 0.393	7.744 ± 1.008	1.570 ± 0.348
	95% CI*	0.011	0.566	0.826	0.017	0.344	0.883	0.305
	**LB**	0.952	2.452	3.504	0.078	1.807	6.861	1.265
	**UP**	0.975	3.584	5.157	0.111	2.495	8.627	1.875
ANN-PSO	**Mean ± SD**	0.891 ± 0.072	5.032 ± 1.409	7.164 ± 1.688	0.159 ± 0.045	3.675 ± 1.131	13.129 ± 4.500	2.541 ± 0.908
	**95% CI**	0.063	1.235	1.480	0.040	0.992	3.945	0.796
	**LB**	0.828	3.797	5.684	0.119	2.683	9.185	1.745
	**UP**	0.954	6.267	8.644	0.198	4.666	17.074	3.337

*RRMSE: Relative Root Mean Square Error, NRMSE: Normalized Root Mean Square Error, CI: Confidence Intervals.

#### Computational efficiency and ablation study

**Fig. 4:**
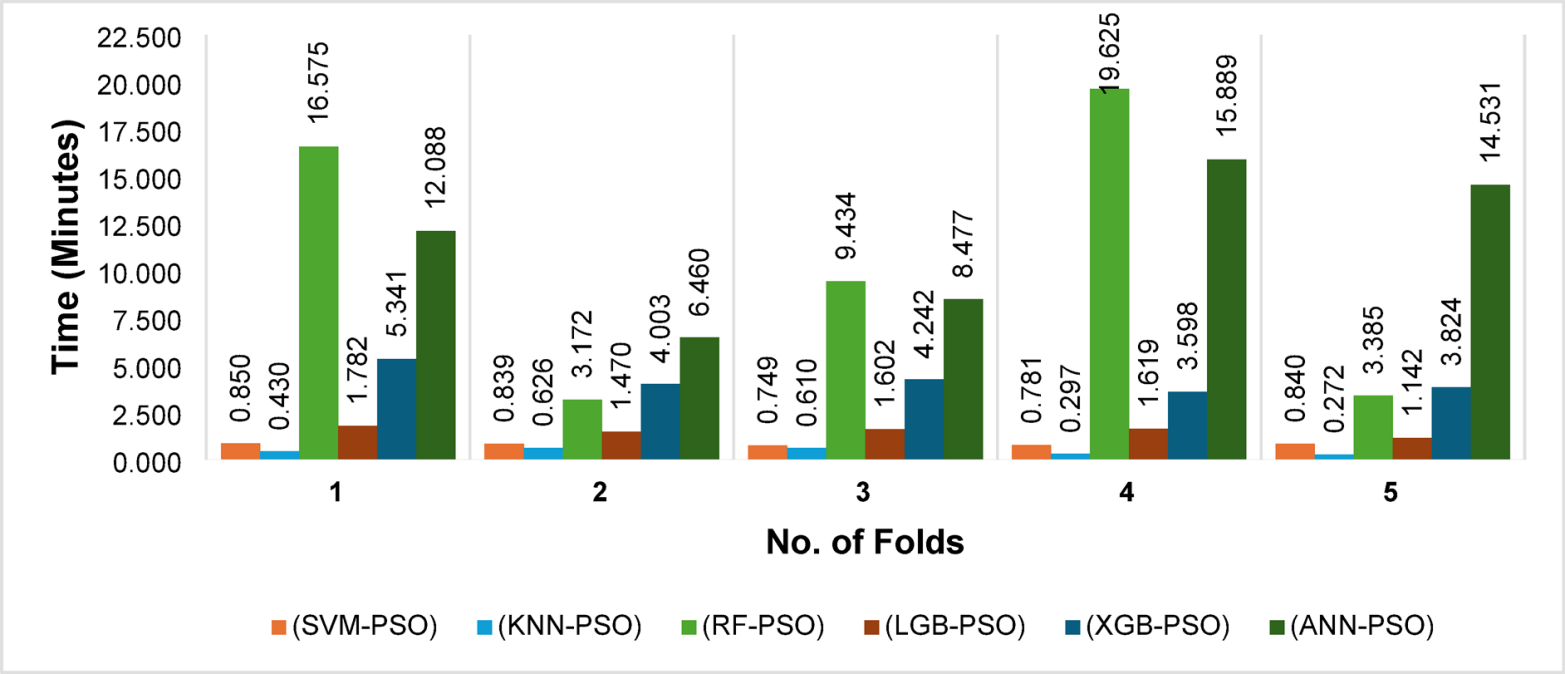
Computational time comparison of PSO-optimized models across 5-fold cross-validation.

The computational efficiency of each PSO-optimized model was examined based on the runtime recorded for each of the five cross-validation folds, as illustrated in Fig. 4. The per-fold timing trends highlight notable contrasts between model simplicity and predictive accuracy. The KNN-PSO and SVM-PSO models were the most time-efficient, completing each fold in average 0.45 min and 0.80 min, respectively. Their minimal parameterization and non-iterative nature contributed to the low computational demand. In contrast, ensemble and deep-learning approaches exhibited higher computational costs due to their iterative optimization and parameter-search processes. The RF-PSO model required the longest time among the tree ensembles (≈ 10.5 min per fold), reflecting the cost of constructing numerous deep decision trees. The LGB-PSO and XGB = PSO models provided a better trade-off between speed and accuracy, consuming 1.52 min and 4.2 min per fold at average, respectively. Although XGB-PSO achieved the highest overall predictive accuracy, its more complex boosting structure and extensive regularization made it slower than LGB-PSO. The ANN-PSO model recorded the longest runtime (≈ 11.5 min per fold) due to repeated backpropagation cycles and PSO-driven weight and hyperparameter optimization, illustrating the expected trade-off between computational complexity and predictive strength.

**Fig. 5:**
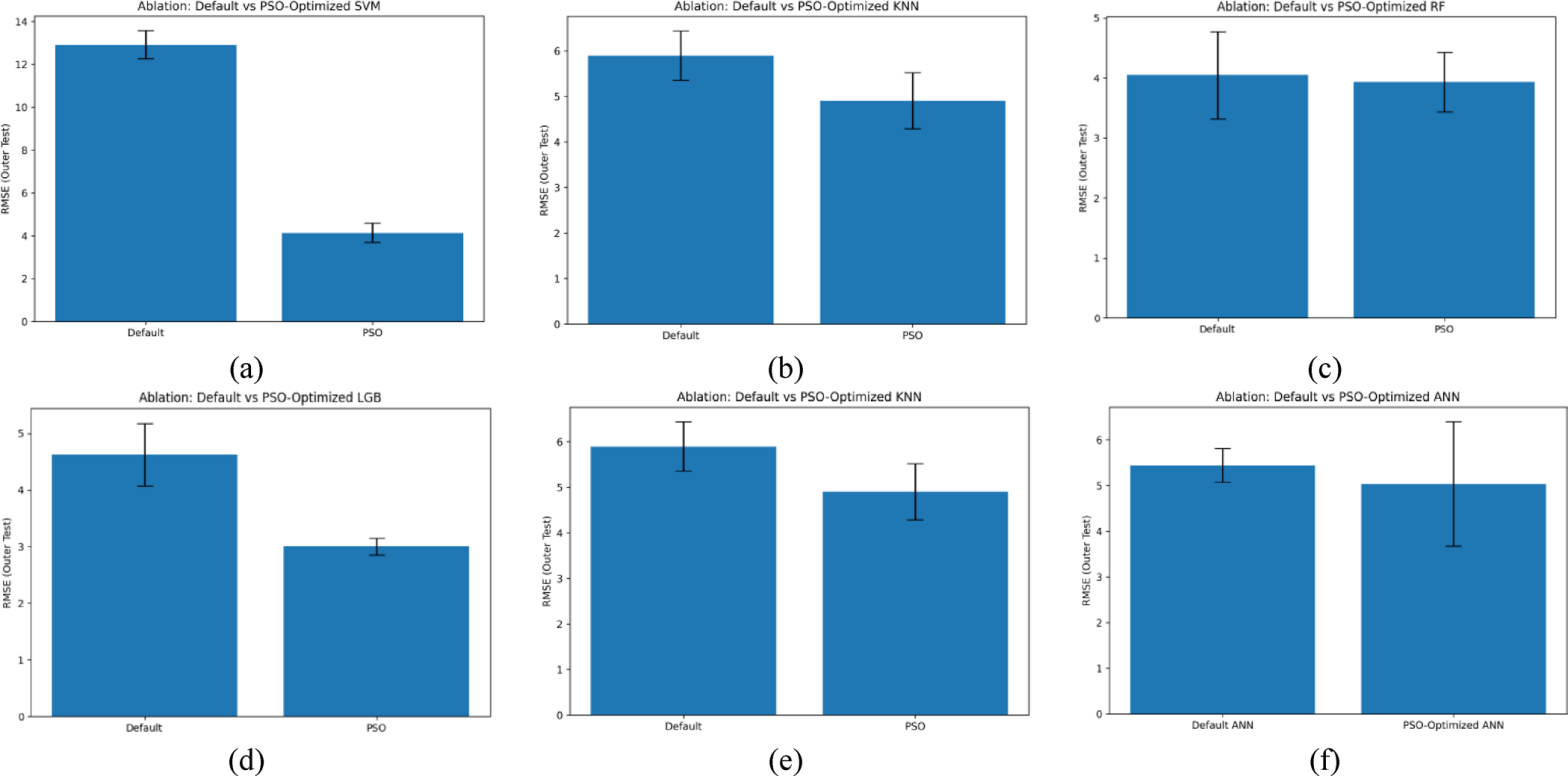
Ablation analysis comparing model performance with and without PSO optimization across six algorithms: (**a**) SVM, (**b**) KNN, (**c**) RF, (**d**) LGB, (**e**) XGB, and (**f**) ANN.

The ablation study (Fig. 5) further highlights how PSO optimization influenced model accuracy relative to their default configurations. All models benefited from PSO-based tuning, but the magnitude of improvement varied according to model type and complexity. The SVM-PSO model exhibited the most substantial gain, with a marked reduction in RMSE compared to its default settings demonstrating that simple models can achieve significant accuracy improvements with efficient metaheuristic tuning at minimal computational cost. In contrast, XGB-PSO, despite being the most accurate model overall, achieved a comparatively smaller incremental improvement due to its already optimized base performance and greater computational expense. LGB-PSO maintained an excellent balance, achieving near-optimal accuracy with modest runtime requirements. Meanwhile, ANN-PSO showed no improvement despite extensive optimization, underscoring the diminishing returns of hyperparameter tuning in complex architectures when computational costs are high.

Overall, these findings emphasize the fundamental balance between computational simplicity and predictive precision. While models such as SVM and KNN deliver rapid, low-cost training, gradient-boosting and neural models like XGB and ANN achieve higher accuracy at the expense of significantly greater computation time. PSO optimization effectively enhances performance across all model families, enabling each to approach its optimal trade-off between simplicity, cost, and predictive reliability.

#### Hyperparameter selection

The hyperparameter optimization of all models was conducted using PSO integrated within the inner folds of the nested cross-validation framework. Within each inner loop, PSO iteratively explored the parameter search space to minimize the mean RMSE across validation folds, ensuring unbiased optimization independent of the outer test data. This systematic tuning process allowed each model to reach its optimal configuration without compromising generalization or inducing overfitting. After completing all five outer folds, the final hyperparameter configuration for each model was determined by averaging the optimized values obtained across folds, ensuring a balanced and representative parameter set for the final model training and evaluation. The optimized hyperparameters obtained from PSO for each algorithm are summarized in Table 4.

**Table 4:** Hyperparameter configurations for the optimized models.

Model	Hyperparameters	Best averaged value
SVM-PSO	Regularization Parameter (C)	877.15
	Epsilon	0.732
	Gamma	0.1641
KNN-PSO	Number of Neighbors	2.4
	Weighting Function	0.8
	Power Parameter for Minkowski Distance	1.012
	Leaf Size	31.8
RF-PSO	Maximum Tree Depth	23
	Number of Estimators (Trees)	150
	Minimum Samples per Split	2
	Minimum Samples per Leaf	1
	Maximum Features	0.325
	Random Seed	42
	Number of Jobs	−1
LGB-PSO	Objective Function	Regression (L2 Loss)
	Maximum Tree Depth	6.6
	Number of Leaves	35.6
	Learning Rate	0.297
	Number of Estimators (Trees)	477
	Minimum Samples per Leaf	7.4
	Maximum Features	0.625
	Random Seed	42
XGB-PSO	Objective Function	Regression (Squared error)
	Maximum Tree Depth	7.6
	Learning Rate	0.120
	Number of Estimators (Trees)	428
	Col sample by tree	0.583
	Row Sampling Ratio (Sub sample)	0.586
	Minimum Child Weight	1.6
	Random Seed	42
ANN-PSO	Model Type	MLP Regressor (Feed-Forward ANN)
	Optimizer (Solver)	Adam
	Activation Function (Hidden Layers)	ReLU
	Activation Function (Output Layer)	Linear
	Hidden Layer Configuration	1 hidden layer with 65 neurons
	Learning Rate (Initial)	0.0121
	Regularization Coefficient (Alpha)	0.000581
	Maximum Iterations (Epochs)	2000
	Early Stopping Patience	30
	Tolerance for Optimization (tol)	1 × 10^− 4^
	Random Seed	42
	Loss Function	Mean Squared Error

### Model performance evaluation

**Fig. 6:**
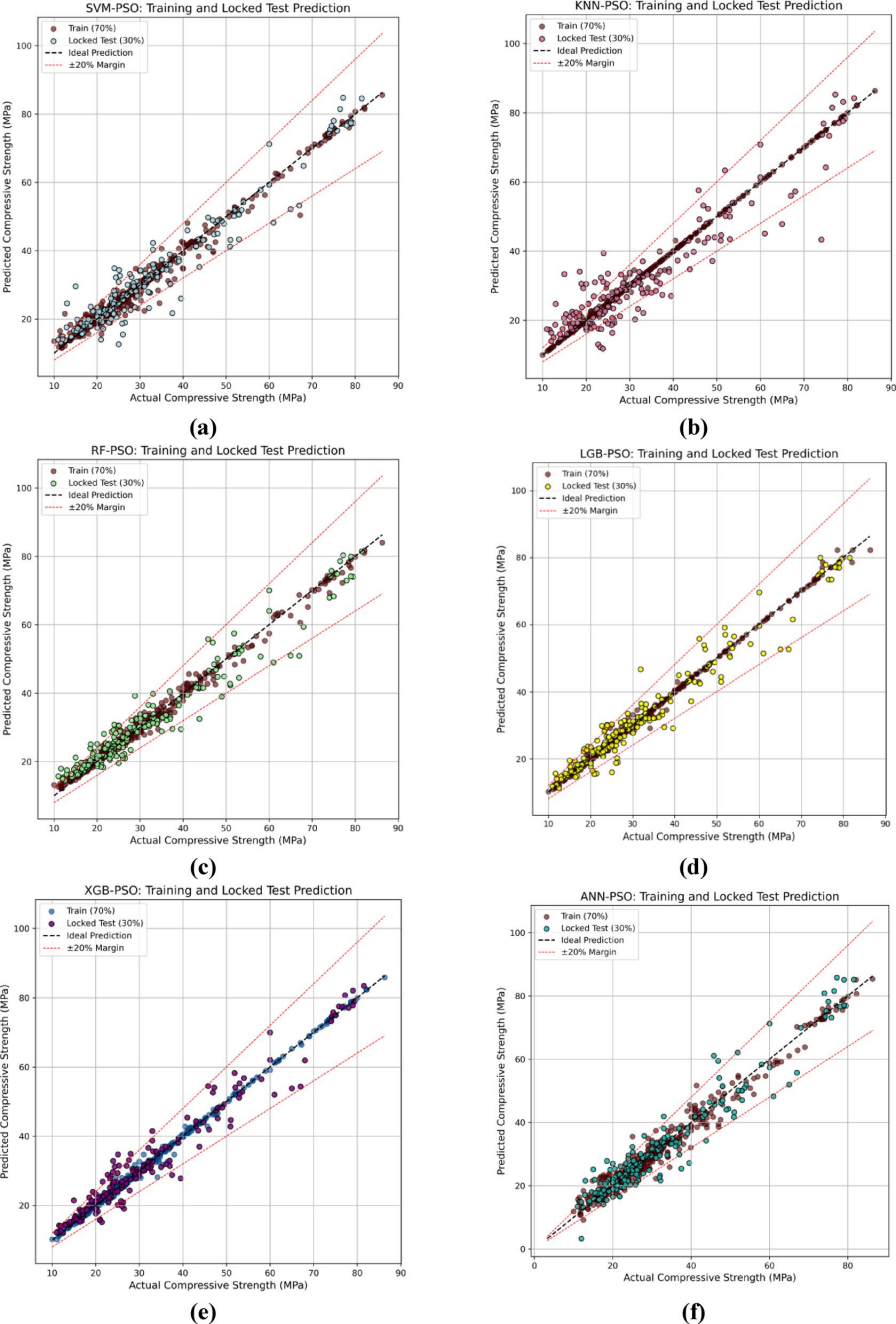
Regression plots of predicted versus actual CS for PSO optimized models: (**a**) SVM-PSO, (**b**) KNN-PSO, (**c**) RF-PSO, (**d**) LGB-PSO, (**e**) XGB-PSO, and (**f**) ANN-PSO.

**Fig. 7:**
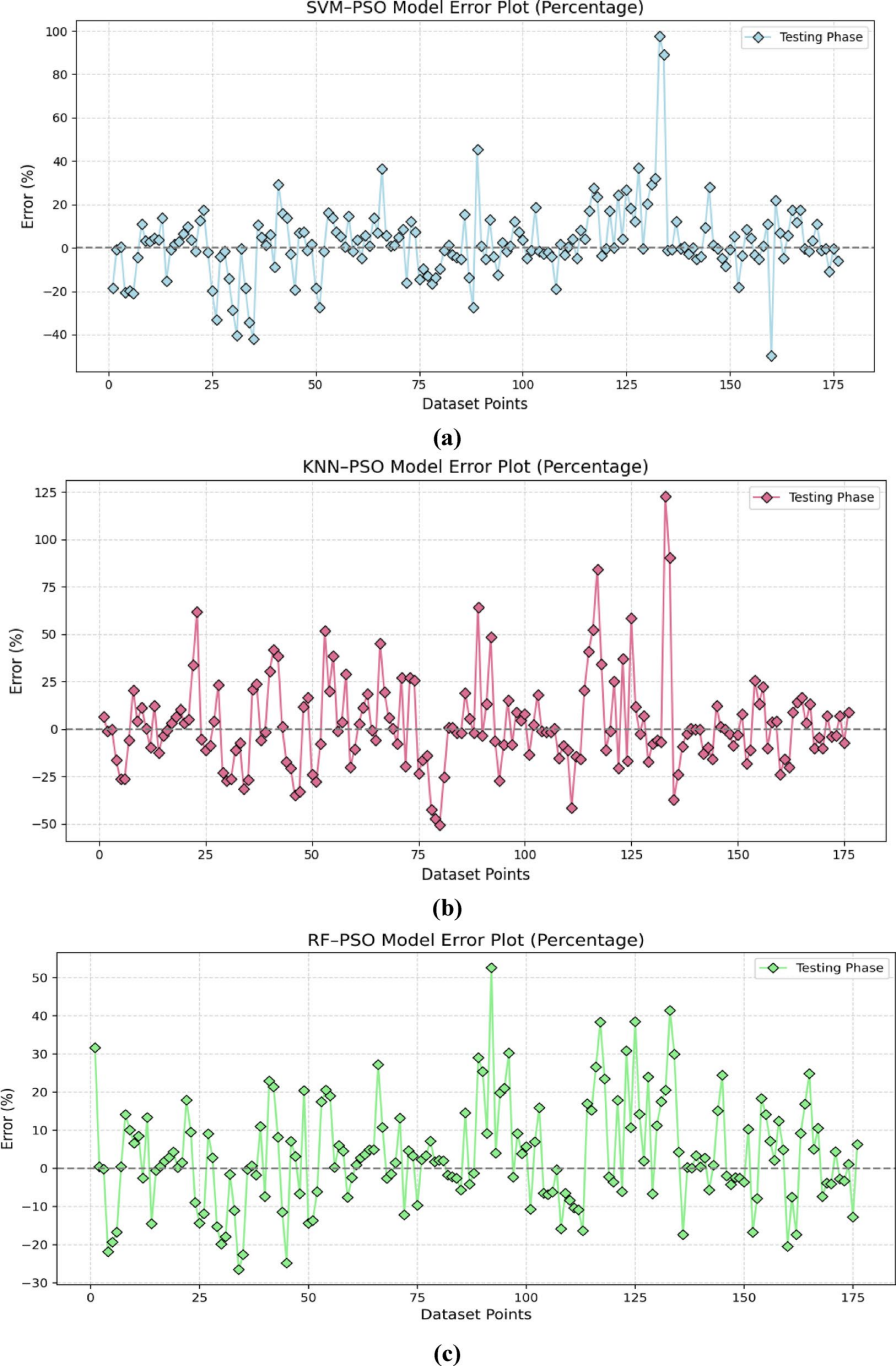

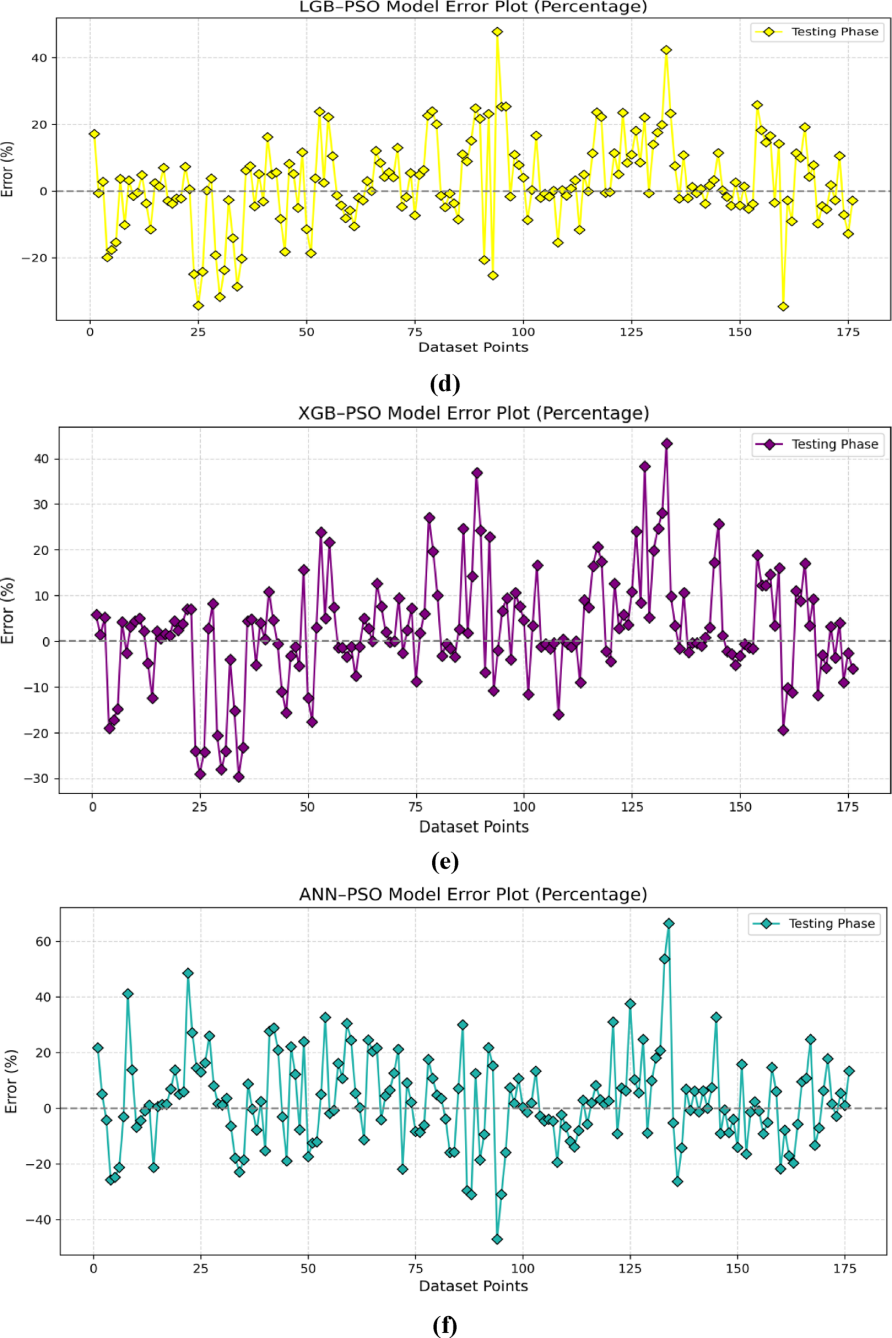
Percentage error distributions for PSO optimized models on the locked test set: (**a**) SVM-PSO, (**b**) KNN-PSO, (**c**) RF-PSO, (**d**) LGB-PSO, (**e**) XGB-PSO, and (**f**) ANN-PSO.

This section addresses Objective 2, which quantifies generalization on the locked 30% test set. Following hyperparameter optimization, the regression plots (Fig. 6(a-f)) illustrate the predictive performance of each model on the held-out test dataset. Most models exhibited strong alignment with the 1:1 prediction line, particularly the XGB-PSO, LGB-PSO, and RF-PSO models. Among all models, the XGB-PSO model exhibited the best generalization performance, achieving the highest coefficient of determination (R^2^ = 0.953) and the lowest error metrics across the locked 30% test set. The LGB-PSO model also demonstrated excellent predictive capability with a comparable R^2^ = 0.950, highlighting its effective balance between bias and variance while having fastest computational efficiency (Sect. 4.1.2). In contrast, KNN-PSO showed relatively weaker generalization (R^2^ = 0.864) and higher error magnitudes, reflecting its sensitivity to data density and local variations. To further understand model behavior, residual error plots were analyzed (Fig. 7(a-f)), depicting the percentage deviation between actual and predicted compressive strength values across all test samples. A generally random distribution of residuals around the zero line was observed, suggesting minimal systematic bias. Notably, XGB-PSO, LGB-PSO, and ANN-PSO displayed tighter and more symmetric residual spreads, confirming their precision and consistency in prediction.

Table 5 summarizes the quantitative evaluation metrics (R^2^, RMSE, MAE, MAPE, NRMSE, and RRMSE) for all PSO-optimized models on the locked test dataset. The XGB-PSO model achieved the lowest RMSE (3.71 MPa), NRMSE (0.113), RRMSE (5.26%), MAE (2.582 MPa), MAPE (8.87%) and Med AE (1.431 MPa), confirming its superior accuracy and stability. The LGB-PSO model followed closely with RMSE = 3.827 MPa and MAPE = 9.07%, exhibiting high predictive reliability. ANN-PSO showed slightly higher absolute errors (RMSE = 4.616 MPa, MAPE = 11.62%) but maintained solid generalization (R^2^ = 0.927). Both SVM-PSO and RF-PSO yielded competitive results with R² values of 0.929 and 0.939, respectively, while KNN-PSO recorded the highest overall errors (RMSE = 6.327 MPa, MAPE = 16.795%).

**Table 5:** Evaluation metrics for ML models using PSO optimized hyperparameters on 30% locked testing dataset.

	Metrics	SVM-PSO	KNN-PSO	RF-PSO	LGB-PSO	XGB-PSO	ANN-PSO
Testing Phase (Locked 30% Set)	R^2^	0.929	0.864	0.939	0.950	0.953	0.927
	RMSE (MPa)	4.540	6.327	4.222	3.827	3.710	4.616
	RRMSE (%)	6.440	8.975	5.989	5.429	5.263	6.548
	NRMSE	0.138	0.193	0.129	0.116	0.113	0.141
	MAE (MPa)	3.046	4.588	3.056	2.661	2.582	3.310
	MAPE (%)	10.972	16.795	10.580	9.071	8.875	11.624
	Med AE (MPa)	1.997	3.367	2.087	1.770	1.431	2.246

### Model explainability and feature interpretability

**Fig. 8:**
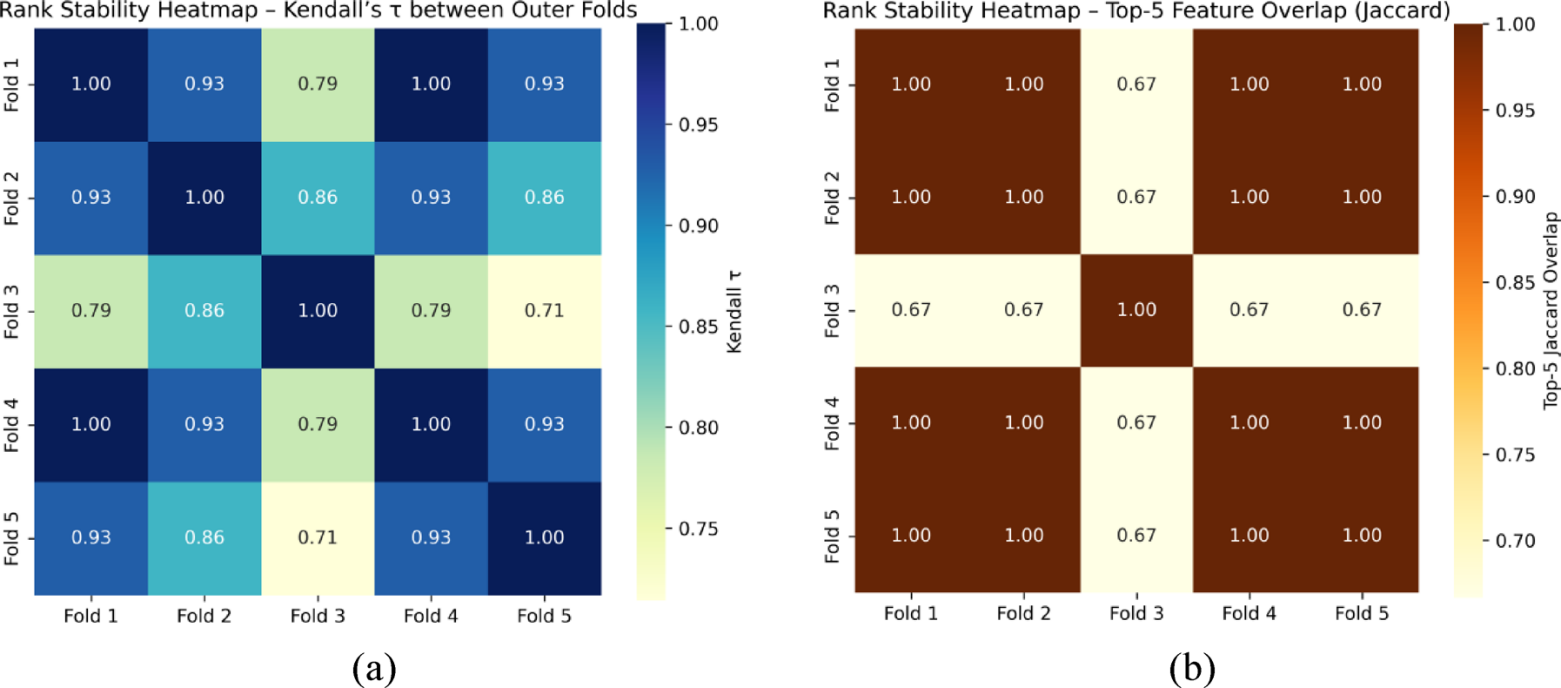
Feature-rank stability across outer folds: (**a**) Kendall’s τ correlation, (**b**) Top 5 Jaccard overlap.

**Fig. 9:**
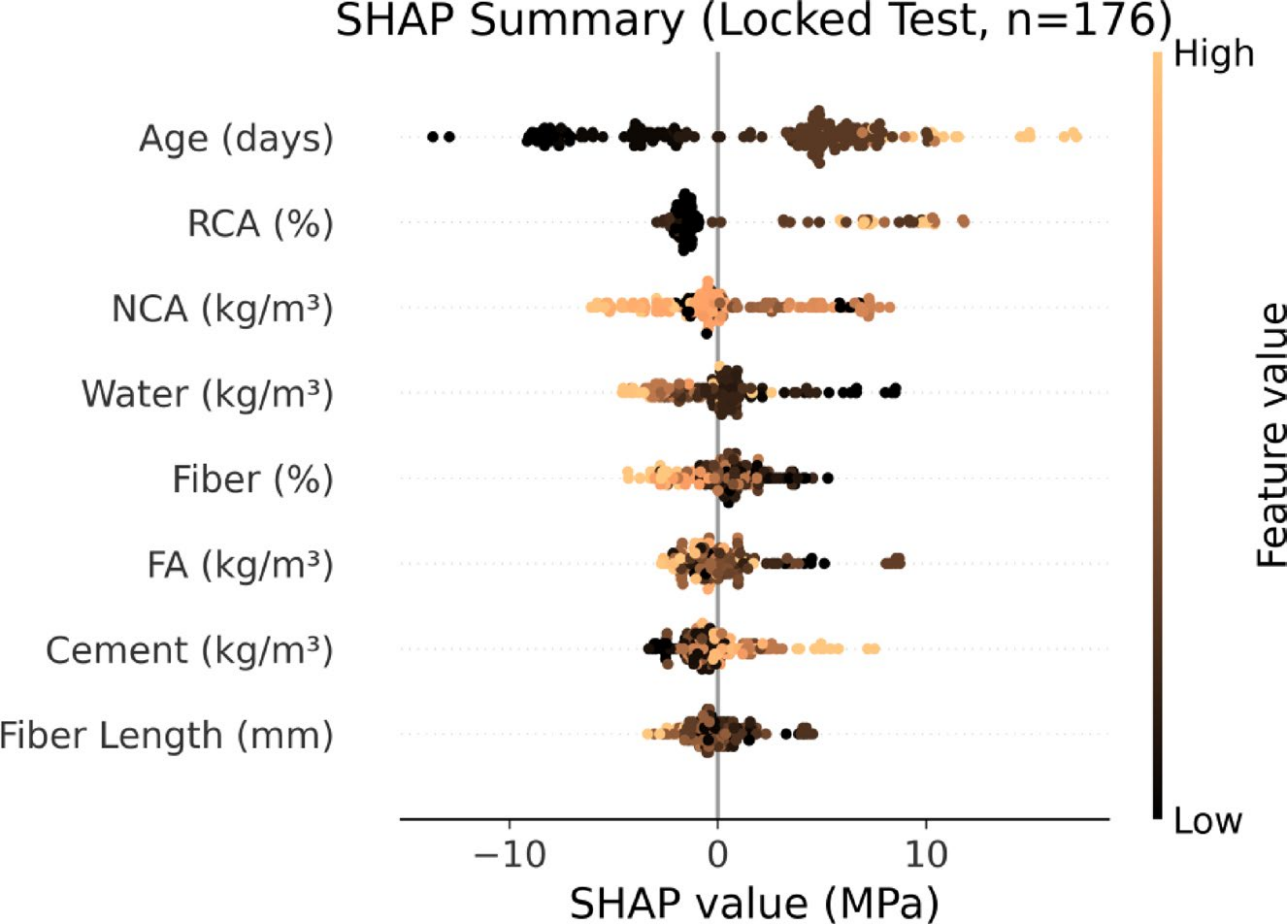
SHAP summary plot showing feature impact on CS predictions for the XGB-PSO model.

**Fig. 10:**
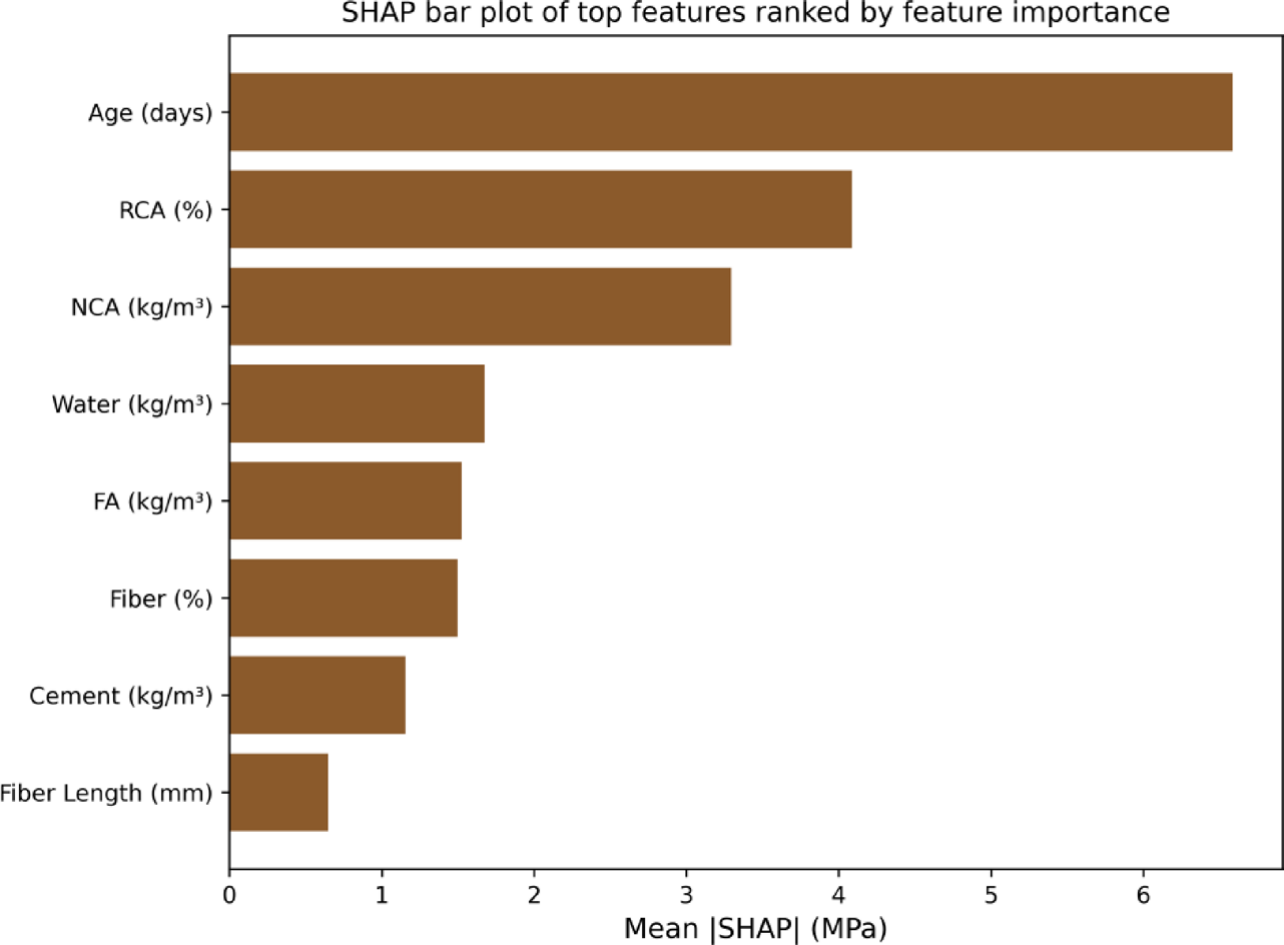
SHAP bar plot of top features ranked by feature importance for the XGB-PSO model.

**Fig. 11:**
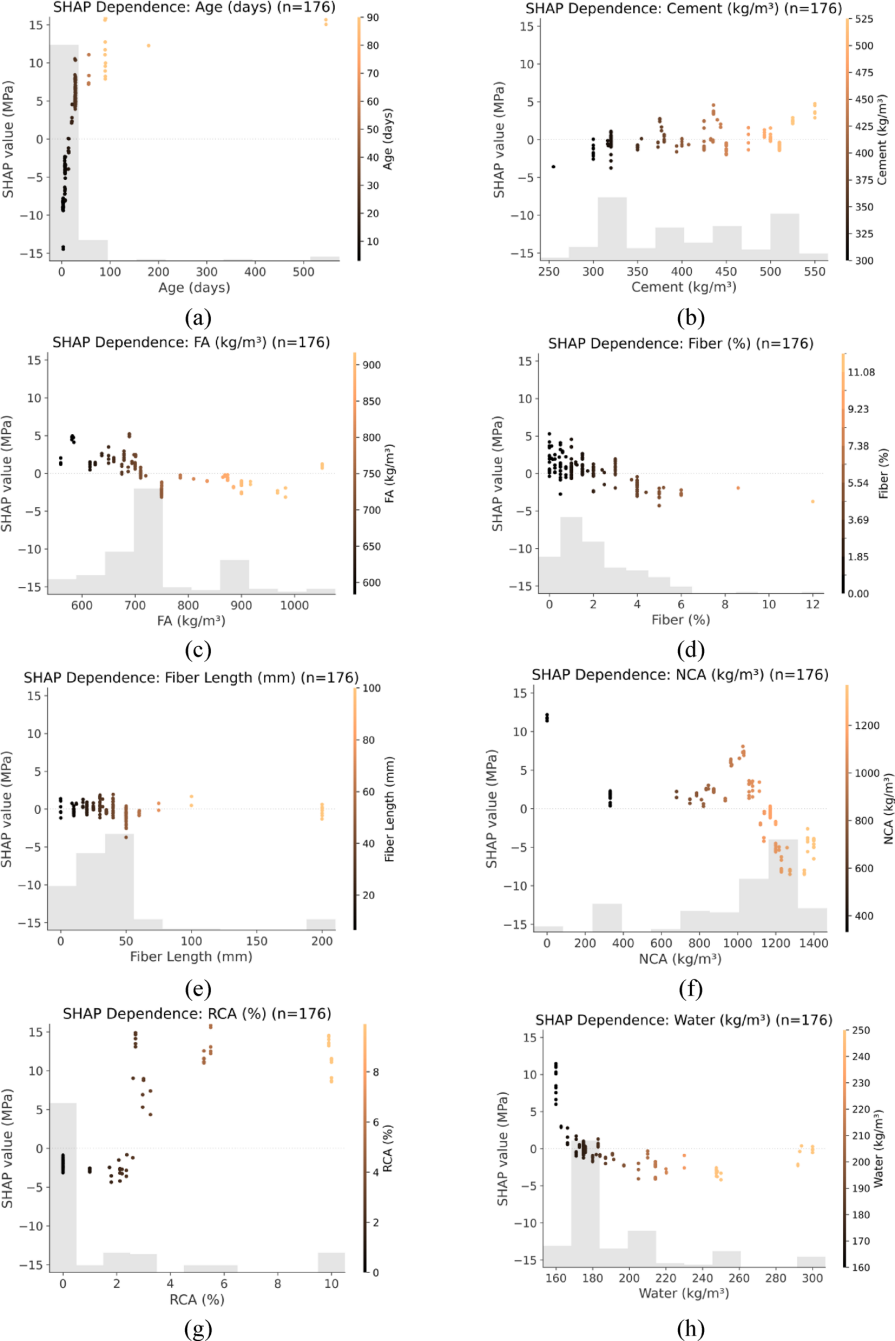
SHAP dependence plots showing how each feature (**a**) Age, (**b**) Cement, (**c**) FA, (**d**) Fiber %, (**e**) Fiber length, (**f**) NCA, (**g**) RCA, (**h**) Water, influences model output.

**Fig. 12:**
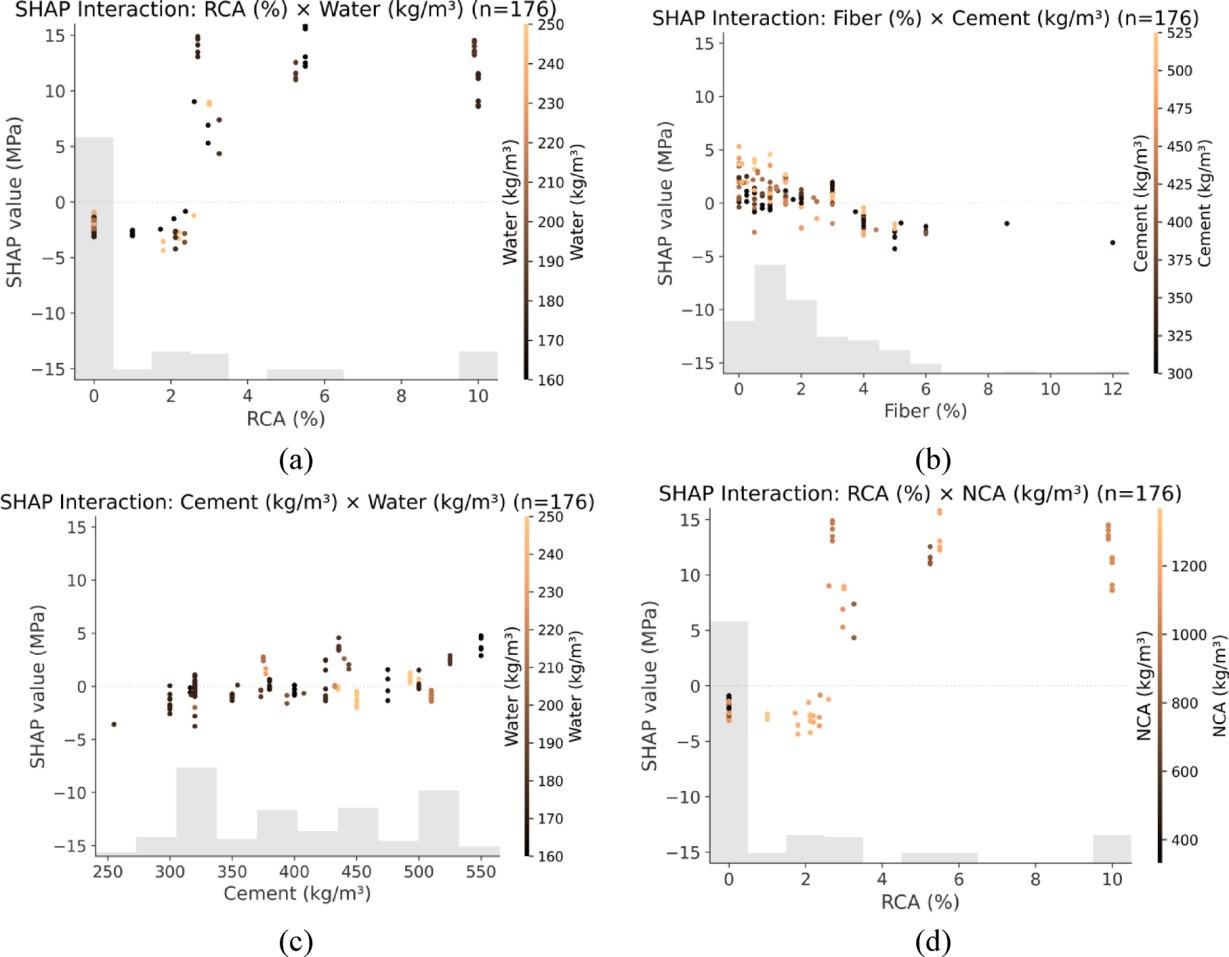
SHAP interaction plots for key feature pairs: (**a**) RCA × Water, (**b**) Fiber content × Cement, (**c**) Cement × Water, (**d**) RCA × NCA.

**Fig. 13:**
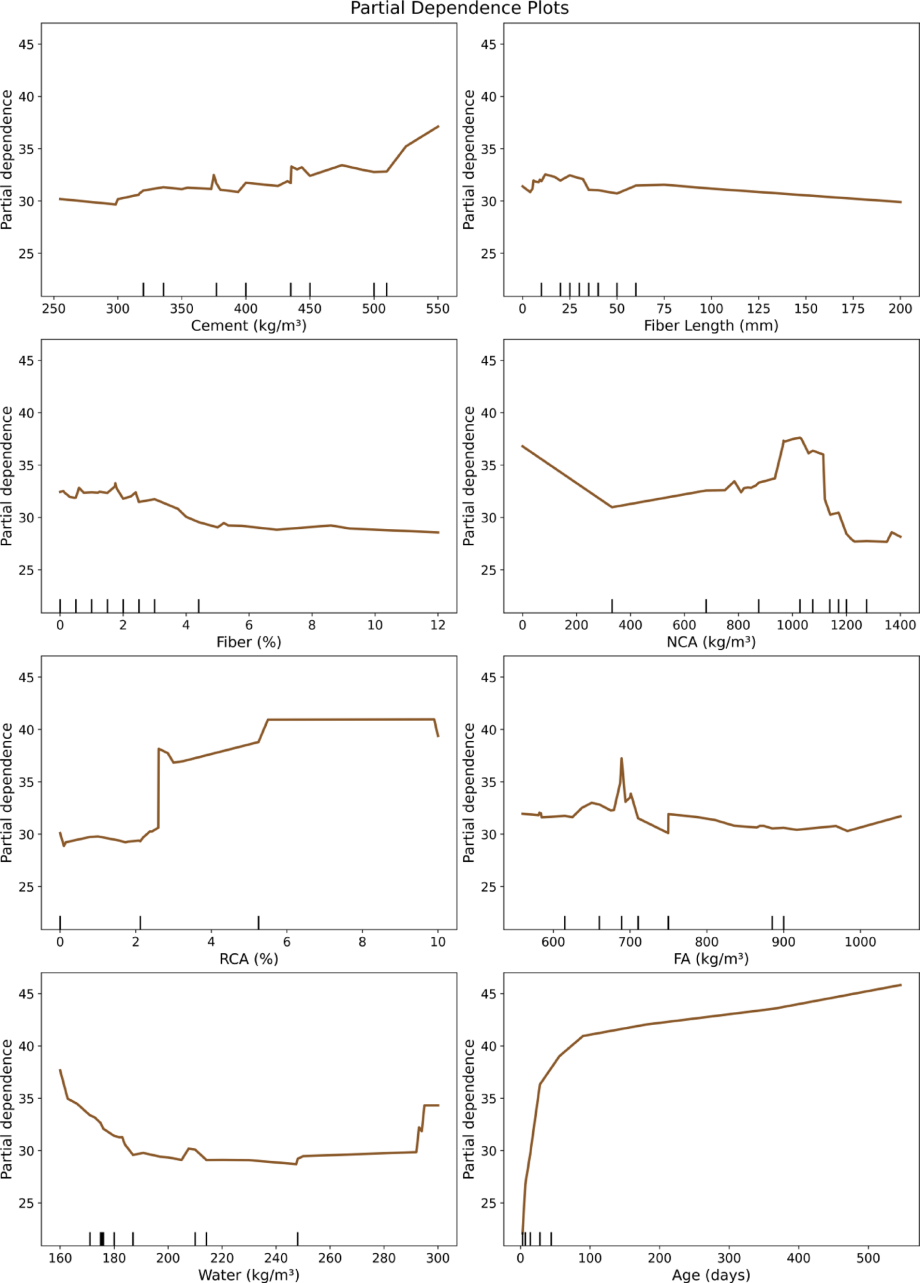
Partial dependence plots illustrating the marginal effect of each input on compressive strength.

**Fig. 14:**
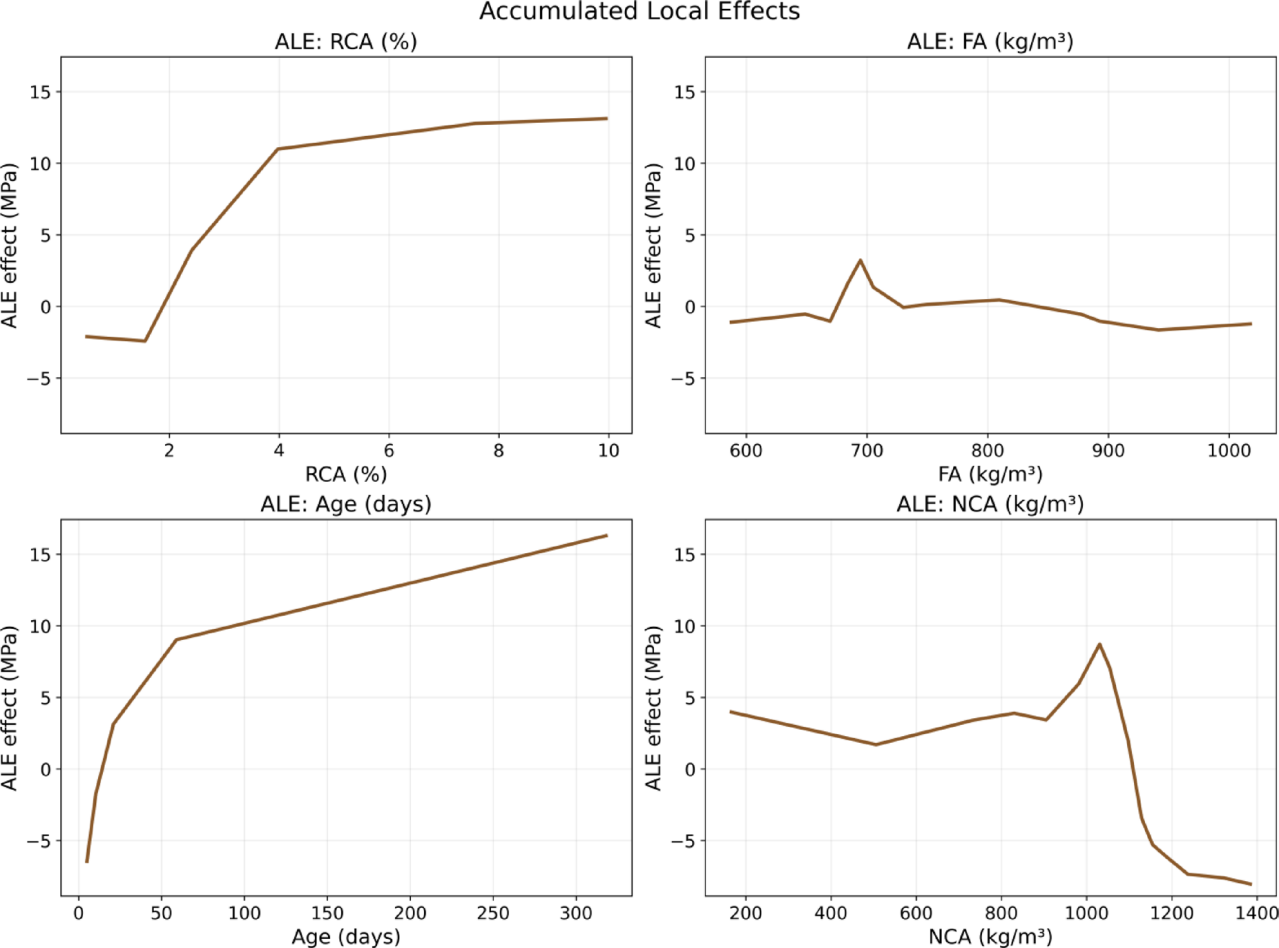
Accumulated Local Effects plots for the top four influential features, RCA (%), FA (kg/m^3^), NCA (kg/m^3^), and Age (days) illustrating localized relationships with compressive strength.

**Fig. 15:**
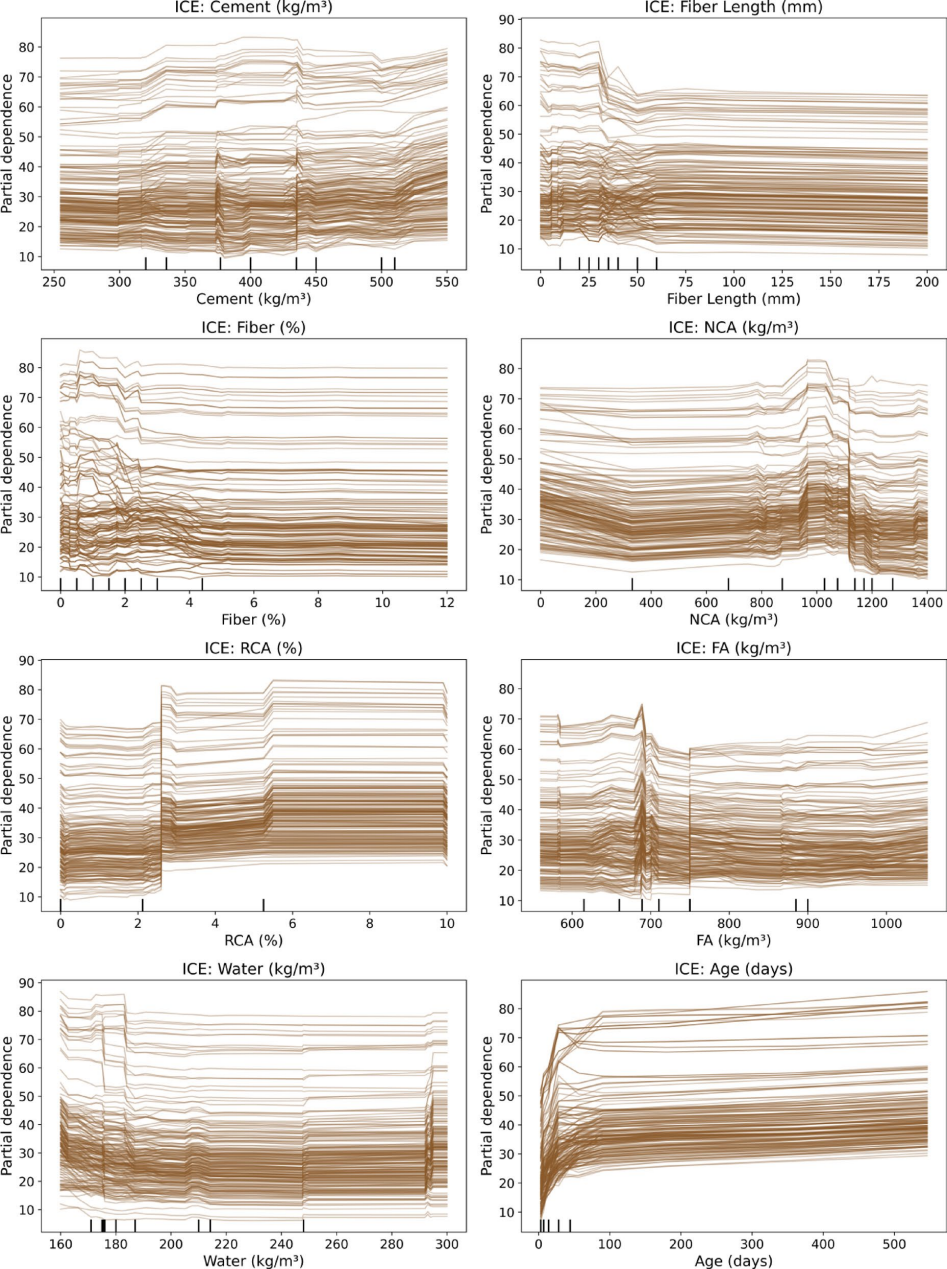
Individual conditional expectation plots showing feature-wise influence on prediction trends.

This section corresponds to Objective 3, focusing on understanding model behavior and identifying influential features. The following analyses use SHAP, PDP, ALE, and ICE to visualize both global and local relationships among mix-design parameters. To ensure consistency, Figs. 8, 9, 10, 11, 12, 13, 14 and 15 were generated using the best-performing model, XGB-PSO.

#### SHAP evaluation and rank stability across folds

The SHAP analysis was performed using the Tree Explainer implementation of the SHAP v0.48.0, which provides exact additive attributes for tree-based models using XGB-PSO. In this study, the 70% training subset was used as the background (reference) dataset for computing SHAP values, while the locked 30% test subset served as the evaluation dataset for model interpretation. All input variables were retained in their original physical units without normalization or scaling, as the XGB algorithm inherently handles features with varying magnitudes. Consequently, the SHAP values are expressed directly in MPa, the same unit as the target compressive strength.

To ensure that the observed feature importance patterns were not specific to a single train-test split, a rank stability assessment was conducted across the five outer cross-validation folds using two complementary measures such as Kendall’s τ correlation and Top 5 Jaccard overlap (Fig. 8). The Kendall’s τ heatmap (Fig. 8 (a)) demonstrated consistently high rank correlation coefficients, ranging from 0.71 to 1.00, with an average of approximately 0.88, confirming that the relative ordering of influential features remained largely consistent across folds. The Top 5 Jaccard overlap matrix (Fig. 8 (b)) further supported this observation, with overlap scores between 0.67 and 1.00, indicating that 4–5 of the top five features were common across folds. Together, these results verify the robustness and stability of the SHAP-based feature ranking, showing that the dominance of age (days), RCA, and NCA are a consistent pattern captured across the model ensemble.

#### Global feature importance assessment

In interpreting the predictive behavior of the optimized XGB-PSO model for compressive strength of CFRC, SHAP was employed. The SHAP summary bee swarm plot (Fig. 9) shows both the magnitude and direction of feature impacts across all locked-test predictions. Age (days) remains the dominant predictor, with SHAP contributions spanning from–12 to + 16 MPa. RCA (%) also shows a strong effect (about − 4 to + 14 MPa), indicating that RCA dosage is associated with either lower or higher predicted strength values. NCA (kg/m³) exhibits a similarly large range (–6 to + 12 MPa), reflecting the role of natural aggregate quality/quantity. Water (kg/m^3^) has a moderate, mostly negative effect at higher contents, ranging about − 3 to + 11 MPa. FA (kg/m^3^) shows a narrower, near-symmetric spread (–4 to + 4 MPa). Cement (kg/m^3^) contributes modestly (–3 to + 4 MPa), while fiber content is generally small and slightly negative at higher dosages (–5 to + 3 MPa). fiber length (mm) has the smallest influence (–2 to + 1 MPa). Ordering by mean |SHAP| places Age first, followed by RCA ≈ NCA, then water/FA, and finally cement, fiber content, and fiber length.

In support of the bee swarm interpretation, the SHAP features importance bar plot (Fig. 10) quantitatively ranked the mean absolute SHAP values. Age recorded the highest mean SHAP value of 6.58, followed by RCA (4.09), NCA (3.30), and water (1.68). Meanwhile, other inputs such as fiber content, FA, cement, and fiber length recorded comparatively lower mean SHAP values (ranging between 0.65 and 1.52), suggesting more modest yet non-negligible effects.

#### SHAP dependence analysis

To better understand the nuanced effects of individual input features on CS predictions, SHAP dependence plots were generated for each variable. Figure 11 illustrate how the SHAP values (representing the marginal contribution of each feature) change with varying feature values, enabling both linear and non-linear interactions to be identified.

Age (days) (Fig. 11a) shows a distinctly positive relationship with SHAP values. Most samples cluster below 100 days, where SHAP contributions increase from about − 15 MPa at very early ages to + 15 MPa for extended curing durations. This pattern confirms the expected strength gain over time due to continued hydration and pozzolanic reactions. Cement (kg/m^3^) (Fig. 11b) exhibits a moderate upward trend, with SHAP values ranging approximately from − 5 to + 6 MPa as cement content increases from ≈ 300 to 520 kg/m^3^. Higher cement dosages consistently enhance predicted strength, reflecting the densification and binder improvement of the matrix. For FA (kg/m^3^) (Fig. 11c), the relationship is slightly inverse at higher contents: SHAP values decline from + 4 MPa toward − 5 MPa as FA content approaches 900–1000 kg/m^3^, implying that excessive fines may induce paste demand or reduce packing efficiency.

Regarding fibers, fiber content (%) (Fig. 11d) shows a generally negative influence as dosage increases, with SHAP values shifting from mildly positive or near-zero contributions at low dosages to approximately − 4 MPa at higher contents. At low fiber levels, particularly below ≈ 2%, coconut fibers may provide limited benefits through micro-crack bridging; however, beyond ≈ 3–4%, the contribution becomes increasingly negative, likely due to reduced workability, fiber clustering, entrapped voids, and weaker matrix compactness. The few high-dosage points should be interpreted cautiously due to their limited frequency, but they consistently indicate strength reduction. Moreover, fiber length (mm) (Fig. 11e) displays minor and mixed contributions, typically within ± 5 MPa. Intermediate lengths (≈ 20–50 mm) yield slightly positive SHAP values, whereas very short or long fibers show neutral or negative effects, possibly due to sub-optimal stress transfer or dispersion.

For NCA (kg/m^3^) (Fig. 11f), SHAP contributions increase up to around 1200 kg/m^3^ (≈ + 13 MPa) before tapering, suggesting that an optimal coarse aggregate volume improves load distribution, while further addition may reduce paste cohesion. RCA (%) (Fig. 11g) shows a bi-modal response i.e., lower contents (≈ 0–2%) correspond to negative SHAP values (down to − 6 MPa), while higher dosages (≈ 8–10%) lead to strong positive contributions (up to + 15 MPa). This indicates that moderate recycled aggregate replacement can be beneficial when bond quality and particle grading are favourable.

Lastly, water (kg/m^3^) (Fig. 11h) presents a clear inverse relationship, with SHAP values declining from + 10 MPa to about − 5 MPa as water increases from ≈ 160 to 300 kg/m^3^. This reinforces the classical water-cement ratio principle: excessive water content elevates porosity and reduces compressive strength. Overall, the dependence plots validate that age, aggregate composition, and cement content are the most decisive parameters, while fiber dosage and water content exhibit strong non-linear penalties beyond their optimal ranges.

To further examine potential feature interactions beyond individual effects, SHAP interaction plots were generated, as shown in Fig. 12(a-d). The interaction between RCA and water (Fig. 12a) revealed that at lower water contents (< 190 kg/m^3^), an increase in RCA led to negative SHAP values (–10 to − 5 MPa), indicating a strength reduction due to inadequate workability and poor ITZ quality. However, at moderate water contents (200–230 kg/m^3^), RCA’s effect turns positive, reaching up to + 15 MPa, suggesting that adequate moisture alleviates stiffness and enhances recycled aggregate bonding. The fiber content (%) × cement (kg/m^3^) interaction (Fig. 12b) shows that low fiber contents, mainly below ≈ 2%, produce near-zero to mildly positive SHAP values, particularly at higher cement contents, suggesting limited fiber matrix contribution. However, as fiber content increases beyond ≈ 3–4%, SHAP values become increasingly negative, reaching about − 4 MPa at higher dosages. This indicates that excessive fiber addition may offset cement-related strength benefits due to fiber clustering, entrapped air, reduced flowability, and weaker matrix compactness. Moreover, the Cement (kg/m^3^) × Water (kg/m^3^) interaction (Fig. 12c) reinforces the water–binder relationship: mixes with higher cement (> 400 kg/m³) and moderate water (≈ 190–210 kg/m^3^) show positive SHAP responses (up to + 5 MPa), while excessive water (> 240 kg/m^3^) consistently reduces contributions to around − 5 MPa. Finally, the RCA (%) × NCA (kg/m^3^) interaction (Fig. 12d) demonstrates complementary aggregate effects. A balanced mix of moderate RCA (≈ 4–6%) and high NCA content (> 1000 kg/m^3^) yields the highest SHAP values (up to + 15 MPa), highlighting optimal packing density and aggregate interlocking. In contrast, low RCA or underfilled NCA combinations correspond to negative SHAP effects (< − 5 MPa). These interactions underline the nonlinear synergy between water balance, binder proportion, and aggregate composition in governing the mechanical response of CFRC.

#### PDP and ALE analysis

To better interpret the marginal influence of each input feature on the predicted compressive strength, PDPs were generated for all eight input variables using the final optimized XGB-PSO model (Fig. 13). These plots illustrate the average effect of each parameter while marginalizing over the others, providing insight into both linear and nonlinear feature responses.

The PDP for age (days) exhibits a distinctly positive relationship with compressive strength. A rapid gain in strength is observed within the early curing period (0–28 days), followed by a gradual increase that stabilizes after approximately 90 days. This pattern reflects the typical strength development behavior driven by cement hydration and supplementary reactions. Similarly, Cement content demonstrates a moderate but consistent positive effect, indicating that higher cement dosage improves binder density and overall strength. In contrast, Water content shows a clear negative influence on compressive strength. As water dosage increases beyond 200 kg/m^3^, the model predicts a steady decline in strength, consistent with the expected weakening effect of higher water-to-binder ratios that reduce matrix compactness.

The RCA (%) variable reveals a slightly nonlinear yet generally positive trend such as strength improves as RCA replacement increases up to around 8–10%, suggesting that the use of treated or quality-controlled RCA can yield satisfactory or even enhanced mechanical performance. Conversely, NCA exhibits a mild negative slope overall, implying that higher natural aggregate proportions do not necessarily improve the mix’s mechanical efficiency compared with its recycled counterpart.

The fiber content (%) feature shows a generally nonmonotonic but predominantly decreasing response. At low dosages, particularly up to approximately 1–2%, the partial dependence remains nearly stable with a slight positive contribution, suggesting that limited fiber addition may provide minor strength benefits. However, beyond about 3–4%, the predicted compressive strength gradually declines, likely due to reduced workability, fiber agglomeration, entrapped voids, and weaker matrix compactness at higher fiber dosages. Fiber length (mm) displays a nearly flat PDP, signifying that variations within the tested length range have minimal isolated effect on strength, possibly because its contribution is governed by interaction with fiber dosage and matrix composition. Finally, FA (kg/m^3^) demonstrates minor fluctuations with a weakly decreasing trend, implying that within the studied range, the fine aggregate proportion exerts only a limited influence on compressive strength.

To complement the global PDP interpretation, ALE plots (Fig. 14) were generated for the top four features, RCA (%), FA (kg/m^3^), NCA (kg/m^3^), and age (days) to examine localized effects without assuming feature independence. The ALE results reaffirm the PDP findings while highlighting more nuanced local sensitivities. The ALE curve for Age shows a consistently positive gradient, indicating strong strength gains with curing, particularly within the early-age range. RCA (%) exhibits a sharp positive influence beyond 2%, confirming its beneficial role in enhancing strength up to the tested range. In contrast, NCA (kg/m^3^) displays a predominantly negative effect, suggesting that higher natural coarse aggregate content may dilute the strength benefits of recycled aggregates. The FA (kg/m^3^) curve remains relatively stable with minor fluctuations, implying limited localized sensitivity to its variation. Overall, the ALE plots confirm that compressive strength is most responsive to changes in RCA (%) and Age (days), while NCA (kg/m^3^) and FA (kg/m^3^) exert subtler influences, in close agreement with the global PDP trends.

#### ICE analysis

To complement the SHAP and PDP interpretations, ICE plots were generated for all input variables to visualize sample-specific responses of the XGB-PSO model (Fig. 15). Unlike PDPs, which average out global effects, ICE plots reveal localized variations across observations, capturing heterogeneity and potential interaction effects in the model’s predictions.

The age (days) variable exhibited the most dominant and consistent positive influence on compressive strength. Nearly all ICE trajectories showed a steep rise in strength up to around 28 days, followed by a gradual stabilization beyond 90 days, with predicted strengths approaching 80–85 MPa for longer curing durations. This reflects the model’s recognition of strength maturity, where early hydration contributes the majority of strength gain before reaching a plateau. The Cement content displayed a moderately positive linear trend across almost all samples. As cement increased from 250 kg/m^3^ to 550 kg/m^3^, predicted compressive strength rose steadily from roughly 30 MPa to 80 MPa, underscoring cement’s consistent role as a primary strength-generating constituent. Conversely, Water content showed a clear negative response. Most ICE lines declined progressively as water increased beyond 200 kg/m^3^, with predictions dropping by up to 15–20 MPa, confirming the detrimental impact of higher water-to-binder ratios that dilute paste density and reduce strength.

For the RCA (%), a distinct stepwise pattern appeared around 2–4%, beyond which most curves rose sharply toward 60–70 MPa. This implies a beneficial threshold where RCA replacement improves performance, likely through optimized packing and the residual pozzolanic reactivity of adhered mortar. In contrast, NCA demonstrated non-monotonic and dispersed ICE trajectories, some increasing while others declined with higher NCA content, suggesting competing effects between aggregate stiffness and interfacial bonding.

The fiber content (%) variable exhibited a mostly negative relationship with compressive strength. Many ICE lines decreased beyond approximately 1–2% fiber content, with high variability across samples, indicating that excessive fiber inclusion may reduce workability and uniformity. Fiber length (mm), however, produced nearly horizontal ICE curves, showing little marginal effect on the model output within the tested range. Finally, FA (kg/m^3^) presented weak, irregular responses, slight dips were observed around 700–800 kg/m^3^, but overall influence on strength remained minimal compared with binder- and aggregate-related parameters.

Overall, the ICE analysis confirms the trends identified through SHAP and PDP interpretations while uncovering localized variability in the model’s behavior. Age, cement, and RCA emerge as the most consistently positive contributors, while water, fiber content, and to a lesser extent FA exhibit predominantly negative or neutral effects. This sample-level insight highlights how mix optimization should emphasize binder efficiency and controlled RCA incorporation while limiting excess water and fiber dosage to achieve higher and more uniform compressive strength.

### Model ranking

**Fig. 16:**
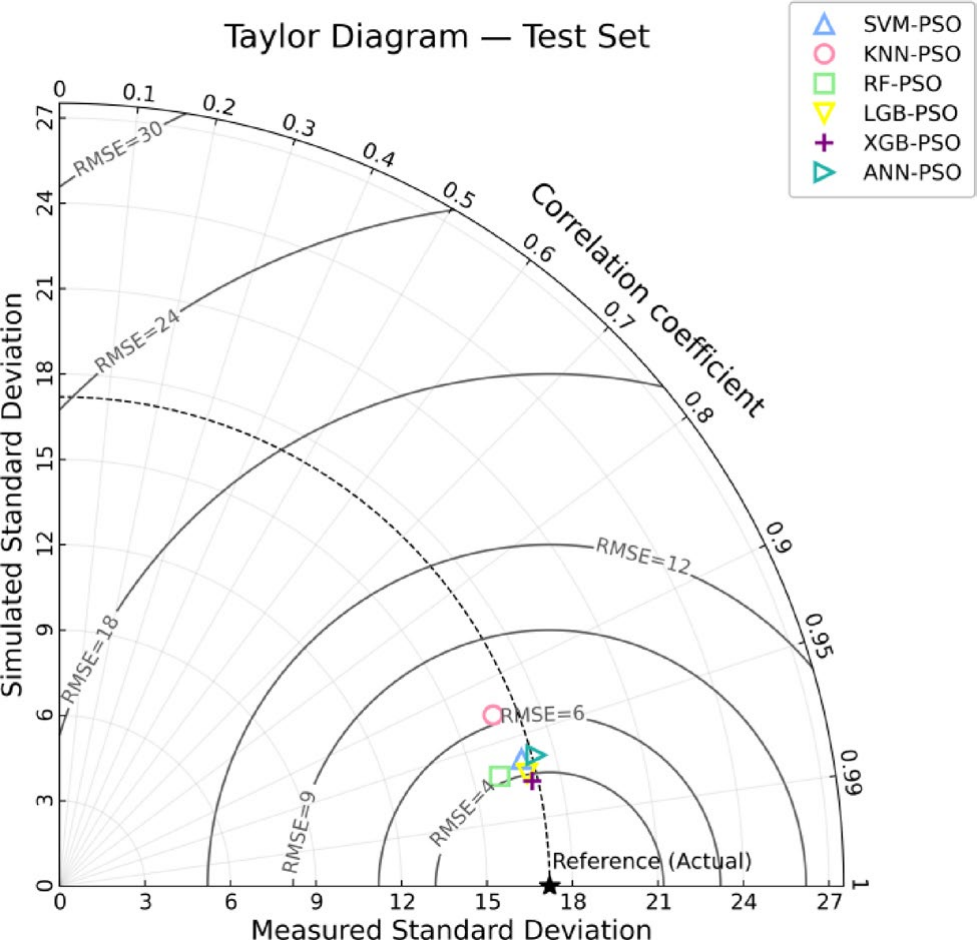
Taylor diagram of model performance on the locked test set (correlation, standard deviation, centered RMSE).

This section corresponds to Objective 4, which evaluates and ranks the optimized learners in terms of accuracy, robustness, and generalization capability. The Taylor diagram (Fig. 16) provides a concise visual comparison of model performance by jointly assessing the correlation coefficient, standard deviation, and centered RMSE relative to the reference (“Actual” star). The ensemble models (XGB-PSO, LGB-PSO, RF-PSO) cluster closest to the reference, confirming their superior predictive accuracy with correlations of 0.976, 0.972, and 0.970, respectively, and standard deviations (15.9–16.9 MPa) closely matching the measured 17.2 MPa. In contrast, SVM-PSO and KNN-PSO show slightly larger deviations (correlations = 0.964 and 0.930), indicating modestly higher error, while ANN-PSO lies farther from the reference, reflecting weaker consistency. This distribution highlights that the PSO-optimized boosted tree models deliver the best balance between accuracy and stability, particularly the XGB-PSO model, which achieved the most favorable combination of correlation, RMSE, and standard deviation^89^. The findings here are in line with studies on similar fiber-reinforced concretes (basalt, polypropylene, etc.), which also report boosted tree models as top performers in strength prediction^31,90^. Overall, the integration of PSO has proven beneficial in optimizing each model’s parameters, leading to improved generalization and making the performance differences more pronounced yet evidence backed by both metrics and Taylor diagram validation.

### Comparative analysis of model strengths and limitations

This section validates the proposed framework by benchmarking its accuracy and generalization against prior ML studies on fiber-reinforced concretes. As summarized in Table 6, earlier investigations typically relied on modest dataset sizes (often 36–103 mixes) and reported best-model performance primarily using either training metrics or limited testing evidence^34–37^. A notable exception is the coconut-fiber study by Kashyap et al^31^., which employed 192 samples and reported strong predictive capability (best model: SVM, CC = 0.952) with sensitivity analysis for interpretation. In comparison, the present study compiles 586 CFRC samples and evaluates PSO-optimized learners using leakage-free validation (nested CV and a locked 30% test split), thereby providing stronger evidence of robustness and out-of-sample generalization. The best-performing model **(**XGB-PSO**)** achieved consistently high predictive performance while maintaining a small train test degradation, which aligns with the general observation that regularized boosting and ensemble learners tend to generalize well in concrete strength prediction tasks^90,91^.

The comparative evidence also supports the dominance of tree-based ensembles and boosted learners in predictive accuracy. Prior CFRC-related work reported that ensemble models can outperform standalone learners; for example, RF achieved the highest test accuracy (R^2^ ≈ 0.935) in coconut-fiber paver blocks, followed by boosting/ANN approaches^37^. Similar trends have been reported in other fiber-reinforced concrete datasets where boosted models (e.g., XGB-type learners) provide high correlation and low error when properly tuned^89^. Nevertheless, optimized “simpler” learners may remain competitive in some settings; for instance, ANN, KNN, and SVM all reported very high performance (R^2^ ≈ 0.98) in a basalt-fiber dataset, with KNN showing favorable MAE^42^. These findings collectively indicate that model performance depends on data scale and feature representation, but that boosted-tree hybrids and robust ensembles most consistently achieve low RMSE/MAPE and stable generalization across datasets^80,89^.

From an implementation perspective, the optimized learners present different trade-offs between accuracy, complexity, and computational efficiency. ANN-PSO and XGB-PSO are comparatively heavier because ANNs require iterative weight updates, while XGB typically builds hundreds of trees, however, XGB benefits from parallel processing and second-order gradient optimization. LGB is often reported to train substantially faster while maintaining competitive accuracy, although its leaf-wise growth strategy may overfit if not properly constrained^92^. In contrast, KNN-PSO is lightweight to deploy because it requires no explicit training, but its prediction cost increases with dataset size due to distance computations. SVM-PSO and RF-PSO occupy a middle ground: SVM training can become demanding for larger datasets, whereas RF training scales with the number of trees and also benefits from parallelization. In the present study, the dataset size remained manageable, making runtime a secondary consideration relative to validated accuracy and generalization; nonetheless, KNN-PSO offers a favorable low-cost option with reasonable accuracy, while the more complex XGB/ANN models provide higher predictive performance at the expense of greater computational demand.

**Table 6:** Comparison of prior ML studies on fiber-reinforced concrete with this study.

Study (year)	Dataset size	Input features	ML models used	Best model (accuracy)	Explainability tools
Kashyap et al^34^.; Jute	103	3 (fiber, % fiber, curing days)	ANFIS, ANN, RF, RT	RF (CC = 0.987 train, 0.924 test)	-
Ahmad et al^35^.; Sisal fiber	68	6 (cement, CA, FA, w/c, % fiber, time)	SVM, GP, ANN, Linear/NLR	ANN (R² = 0.96 train, 0.99 test)	-
Moraes et al^36^.; Babassu coconut	51	6 (cement, FA, w/c, fiber length, % fiber, slump)	ANN (ELM)	ANN (R^2^ = 0.876 test)	-
Kiran et al^37^.; Coconut & CDW	36	4 (cement, FA, CDW%, coconut fiber%)	RSM, SVM, GB, ANN, RF	RF (R² =0.935 test)	-
Onyelowe et al^42^.; Basalt fiber	309	10 (cement, fly ash, water, SP, CA, FA, age, fiber length, fiber diameter, % fiber)	ANN, KNN, SVM, DT, RF	KNN (R² =0.99 train, 0.98 test)	Correlation matrix, sensitivity analysis
Kashyap et al^31^.; Coconut fiber	192	8 (CF%, cement, water, CA, FA, fiber length, SP, curing days)	LR, SVM, ANN, Meta_Bagging-SVM, Meta_AddReg-SVM, M5P, RF	SVM (CC = 0.952 test)	Sensitivity Analysis
This Study	586	8 (CF%, cement, water, CA, FA, fiber length, SP, curing days)	SVM-PSO, KNN-PSO, RF-PSO, LGB-PSO, XGB-PSO, ANN-PSO	XGB-PSO (R^2^ = 0.963 train, 0.953 test)	SHAP, PDP, ALE, ICE, Taylor Diagram

*CC = Correlation Coefficient.

## Broader implications and future perspectives

Practical implications.

The explainability analysis (SHAP, PDP, ALE, and ICE) provided valuable insights into how each input parameter influences the compressive strength of CFRC. The following practical guidance can be drawn from the model’s interpretability results:

- **Curing age**: Strength increases rapidly up to 28 days and continues moderately thereafter, supporting 28-day strength as a practical benchmark.
- **RCA content**: RCA shows a positive influence up to about 25–30%, after which strength tends to decrease, indicating that partial RCA replacement can enhance sustainability without compromising strength.
- **Cement content**: Cement has a strong positive influence, with the most efficient range around 400–480 kg/m^3^; beyond this, gain becomes minimum.
- **Fiber dosage**: Fiber improves performance up to about 1.0–1.5%, while higher contents (≈ > 2%) reduce strength, likely due to dispersion and workability issues.
- **Fiber length**: The most favourable range is 40–50 mm; longer fibers (≈ > 60 mm) may cause entanglement and voids.
- **Water content**: Higher water generally reduces strength; moderate water levels (≈ 160–180 kg/m^3^) support workability with limited strength loss.

Overall, these findings provide practical ranges that balance strength performance and sustainability (e.g., RCA and natural fiber use) for CFRC mix proportioning.

### Future recommendations

Based on the current findings and limitations, the following future directions are suggested:

- Adding domain-informed features (e.g., water-binder ratio, paste volume, aggregate-binder ratio).
- Expanding the dataset with more diverse materials, curing regimes, binder systems, and hybrid fiber combinations.
- Incorporating uncertainty quantification (e.g., prediction intervals or Bayesian ensembles) for decision support.
- Performing external/prospective validation using independent laboratory or field datasets.
- Extending PSO-based modelling toward multi-objective optimization (cost, sustainability, durability, and strength).
- Exploring complementary interpretability approaches (e.g., LIME or surrogate models) alongside SHAP-based analysis.

### Research limitations

While the PSO-optimized machine-learning framework demonstrated strong predictive capability for CFRC compressive strength, several limitations should be acknowledged. The compiled dataset is heterogeneous because it was assembled from multiple published studies that differ in raw material sources, mix proportioning procedures, curing regimes, specimen preparation, and testing conditions. Although such diversity can improve model exposure to broader mix variations, it may also introduce inter-study inconsistencies that affect absolute prediction precision. In addition, the reliance on literature-derived data rather than controlled laboratory experimentation means that variability in data quality, reporting completeness, and experimental rigor cannot be fully standardized, and the dataset may be influenced by publication bias if atypical or low-performance results are underreported. The feature set was also limited to eight mix parameters and did not explicitly include chemical admixtures (e.g., superplasticizers), workability indicators (e.g., slump/flow), or additional mix-design descriptors that can influence fiber dispersion and strength development. Finally, although nested cross-validation and a locked test split were used to assess generalization within the compiled dataset, external validation using independent laboratory or field datasets remains necessary to confirm transferability to new CFRC mixtures and practical conditions.

## Conclusion

This study developed and evaluated six PSO optimized machine learning models, SVM, KNN, RF, LGB, XGB, and ANN to predict the compressive strength of CFRC. The analysis followed four structured objectives i.e., comparison of model performance, quantification of generalization on a held-out test set, interpretation of key mix-design drivers, and assessment of robustness using Taylor diagrams. The key findings are presented below.

- On the locked 30% test set, XGB-PSO achieved the highest predictive performance (R^2^ = 0.953, RMSE = 3.710 MPa, MAPE = 8.875%, MedAE = 1.431 MPa).
- LGB-PSO ranked second (R^2^ = 0.950, RMSE = 3.827 MPa, MAPE = 9.071%) and provided faster computation than XGB-PSO, indicating an effective accuracy–efficiency balance.
- SVM-PSO produced balanced performance (R^2^ = 0.929) with low runtime, while RF-PSO achieved reliable accuracy (R^2^ = 0.939) with higher computational cost due to large ensembles.
- ANN-PSO yielded moderate accuracy (R^2^ = 0.927) with the highest runtime due to iterative training and PSO-based tuning, whereas KNN-PSO showed the lowest accuracy (R^2^ = 0.864) and highest error (RMSE = 6.327 MPa).
- SHAP-based interpretation identified age and RCA content as dominant predictors, while excessive fiber content and water content reduced strength; PDP, ALE, and ICE analyses confirmed these nonlinear effects and interactions.
- The Taylor diagram analysis indicated the strongest agreement for XGB-PSO in terms of correlation, centered RMSE, and standard deviation, supporting its stability and generalization on the locked test set.

The proposed PSO-optimized and explainable framework enables accurate and transparent CFRC strength estimation and can reduce trial-and-error in mix design, supporting more efficient and sustainability-oriented concrete development.

## Supplementary Information

Below is the link to the electronic supplementary material.

Supplementary Material 1

## Data Availability

The data supporting the findings of this study is made available as a supplementary file. The accompanying code, including the environment file and a reproducible entry-point script is openly accessible in the following public repository: https://github.com/Ssarmadmahmood/ML-Coconut-Fiber-Reinforced-Concrete.
