# Supplementary material for: Compressive strength prediction of coconut fiber reinforced concrete using PSO optimized explainable machine learning
Source: Sci Rep. 2026 Jun 5;16:20776. doi: 10.1038/s41598-026-56658-4 (PMC13338410; doi:10.1038/s41598-026-56658-4)
Supplement: Supplementary file 1 — Supplementary Material 1 [file 41598_2026_56658_MOESM1_ESM.docx]

**Explainable and PSO Optimized Machine Learning Framework for Predicting the Compressive Strength of Coconut Fiber Reinforced Concrete using SHAP, PDP, ALE and ICE**

**Supplementary File**

1. **Data Source References with Access Links**

The following references detail the original sources of the dataset used in this study. Each source is publicly accessible, and the links provided lead to where the data can be found.

| **Paper ID** | **Reference** | **DOI** | **No of Data points** |
| --- | --- | --- | --- |
| 1 | [39] | <https://doi.org/10.17577/IJERTV4IS060939> | 05 |
| 2 | [40] | <https://doi.org/10.3390/MA13051075> | 12 |
| 3 | [41] | <https://doi.org/10.1088/2053-1591/AC10D3> | 21 |
| 4 | [18] | <https://doi.org/10.1016/J.MATPR.2018.10.173> | 10 |
| 5 | [42] | <https://doi.org/10.1016/J.CONBUILDMAT.2012.09.002> | 25 |
| 6 | [43] | <https://doi.org/10.3390/MA11091726> | 168 |
| 7 | [44] | <https://www.irjet.net/archives/V6/i5/IRJET-V6I5433.pdf> | 12 |
| 8 | [45] | <https://doi.org/10.3390/APP122211734> | 11 |
| 9 | [46] | <https://doi.org/10.1007/S11356-022-21608-W/METRICS> | 08 |
| 10 | [47] | <https://doi.org/10.1016/J.CONBUILDMAT.2016.12.092> | 04 |
| 11 | [48] | <https://doi.org/10.1016/J.CONBUILDMAT.2019.01.103> | 02 |
| 12 | [49] | <https://doi.org/10.1038/s41598-025-92227-x> | 12 |
| 13 | [50] | <https://doi.org/10.3390/FIB10110096> | 30 |
| 14 | [51] | <https://doi.org/10.1016/J.CLET.2025.100983> | 18 |
| 15 | [52] | <https://doi.org/10.1016/J.CONBUILDMAT.2025.140262> | 04 |
| 16 | [53] | <https://doi.org/10.1016/J.ASEJ.2024.102666> | 15 |
| 17 | [54] | <https://doi.org/10.1016/J.MATPR.2021.10.370> | 28 |
| 18 | [55] | <https://doi.org/10.1016/J.CONBUILDMAT.2015.12.037> | 12 |
| 19 | [56] | [(PDF) Coconut Fibre Reinforced Concrete](https://www.researchgate.net/publication/275407239_Coconut_Fibre_Reinforced_Concrete) | 08 |
| 20 | [57] | <https://naujcve.com/index.php/NAUJCVE/article/view/37> | 20 |
| 21 | [58] | <https://iaeme.com/Home/article_id/IJCIET_09_01_040> | 18 |
| 22 | [59] | <https://doi.org/10.3390/BUILDINGS12091450> | 15 |
| 23 | [19] | <https://doi.org/10.1016/J.CONBUILDMAT.2016.09.118> | 20 |
| 24 | [60] | <https://doi.org/10.1016/j.heliyon.2024.e24313> | 17 |
| 25 | [61] | <https://doi.org/10.1016/j.heliyon.2024.e39773> | 15 |
| 26 | [62] | <https://doi.org/10.1155/2024/8275876> | 18 |
| 27 | [63] | <https://doi.org/10.22034/JCEMA.2021.143869> | 10 |
| 28 | [64] | <https://doi.org/10.1016/J.CONBUILDMAT.2019.07.232> | 08 |
| 29 | [65] | <https://doi.org/10.3390/FIB8010005> | 32 |
| 30 | [66] | <https://doi.org/10.3390/MA15072616> | 08 |
| **Total** | | | **586** |

1. **Dataset**

| **Paper ID** | **Cement**  **(kg/m^3^)** | **Fiber Length** | **Fiber (%)** | **NCA**  **(kg/m^3^)** | **RCA**  **(%)** | **FA**  **(kg/m^3^)** | **Water**  **(kg/m^3^)** | **Age (Days)** | **Compressive Strength (MPa)** |
| --- | --- | --- | --- | --- | --- | --- | --- | --- | --- |
| 1 | 320 | 0 | 0 | 821 | 3.26 | 968 | 182 | 28 | 28.8 |
|  | 320 | 50 | 0.5 | 821 | 3.26 | 968 | 182 | 28 | 29.12 |
|  | 320 | 50 | 0.75 | 821 | 3.26 | 968 | 182 | 28 | 31.01 |
|  | 320 | 50 | 1 | 821 | 3.26 | 968 | 182 | 28 | 32.38 |
|  | 320 | 50 | 1.25 | 821 | 3.26 | 968 | 182 | 28 | 32.9 |
| 2 | 525 | 25 | 0.5 | 785 | 5.25 | 785 | 184 | 28 | 52 |
|  | 525 | 25 | 1 | 785 | 5.25 | 785 | 184 | 28 | 54 |
|  | 525 | 25 | 1.5 | 785 | 5.25 | 785 | 184 | 28 | 56 |
|  | 525 | 25 | 2 | 785 | 5.25 | 785 | 184 | 28 | 48 |
|  | 525 | 50 | 0.5 | 785 | 5.25 | 785 | 184 | 28 | 67 |
|  | 525 | 50 | 1 | 785 | 5.25 | 785 | 184 | 28 | 65 |
|  | 525 | 50 | 1.5 | 785 | 5.25 | 785 | 184 | 28 | 61 |
|  | 525 | 50 | 2 | 785 | 5.25 | 785 | 184 | 28 | 41 |
|  | 525 | 75 | 0.5 | 785 | 5.25 | 785 | 184 | 28 | 60 |
|  | 525 | 75 | 1 | 785 | 5.25 | 785 | 184 | 28 | 57 |
|  | 525 | 75 | 1.5 | 785 | 5.25 | 785 | 184 | 28 | 53 |
|  | 525 | 75 | 2 | 785 | 5.25 | 785 | 184 | 28 | 43 |
| 3 | 425 | 0 | 0 | 1275 | 2.12 | 625 | 180 | 7 | 12.1 |
|  | 425 | 10 | 0.5 | 1275 | 2.12 | 625 | 180 | 7 | 12.5 |
|  | 425 | 10 | 1 | 1275 | 2.12 | 625 | 180 | 7 | 13.8 |
|  | 425 | 10 | 1.5 | 1275 | 2.12 | 625 | 180 | 7 | 14.2 |
|  | 425 | 10 | 2 | 1275 | 2.12 | 625 | 180 | 7 | 15.7 |
|  | 425 | 10 | 2.5 | 1275 | 2.12 | 625 | 180 | 7 | 14.1 |
|  | 425 | 10 | 3 | 1275 | 2.12 | 625 | 180 | 7 | 13.7 |
|  | 425 | 0 | 0 | 1275 | 2.12 | 625 | 180 | 14 | 16.8 |
|  | 425 | 0 | 0.5 | 1275 | 2.12 | 625 | 180 | 14 | 17 |
|  | 425 | 0 | 1 | 1275 | 2.12 | 625 | 180 | 14 | 18 |
|  | 425 | 0 | 1.5 | 1275 | 2.12 | 625 | 180 | 14 | 19.3 |
|  | 425 | 0 | 2 | 1275 | 2.12 | 625 | 180 | 14 | 20 |
|  | 425 | 0 | 2.5 | 1275 | 2.12 | 625 | 180 | 14 | 18.4 |
|  | 425 | 0 | 3 | 1275 | 2.12 | 625 | 180 | 14 | 17.6 |
|  | 425 | 0 | 0 | 1275 | 2.12 | 625 | 180 | 28 | 18.8 |
|  | 425 | 0 | 0.5 | 1275 | 2.12 | 625 | 180 | 28 | 19.7 |
|  | 425 | 0 | 1 | 1275 | 2.12 | 625 | 180 | 28 | 20.8 |
|  | 425 | 0 | 1.5 | 1275 | 2.12 | 625 | 180 | 28 | 22.3 |
|  | 425 | 0 | 2 | 1275 | 2.12 | 625 | 180 | 28 | 22.7 |
|  | 425 | 0 | 2.5 | 1275 | 2.12 | 625 | 180 | 28 | 21 |
|  | 425 | 0 | 3 | 1275 | 2.12 | 625 | 180 | 28 | 20.1 |
| 4 | 443.7 | 32 | 0 | 1114.34 | 0 | 637.36 | 190.8 | 7 | 32.1 |
|  | 443.7 | 32 | 0.5 | 1114.34 | 0 | 637.36 | 190.8 | 7 | 30 |
|  | 443.7 | 32 | 1 | 1114.34 | 0 | 637.36 | 190.8 | 7 | 35.4 |
|  | 443.7 | 32 | 1.5 | 1114.34 | 0 | 637.36 | 190.8 | 7 | 37.5 |
|  | 443.7 | 32 | 2 | 1114.34 | 0 | 637.36 | 190.8 | 7 | 38.6 |
|  | 443.7 | 32 | 0 | 1114.34 | 0 | 637.36 | 190.8 | 28 | 37.5 |
|  | 443.7 | 32 | 0.5 | 1114.34 | 0 | 637.36 | 190.8 | 28 | 35 |
|  | 443.7 | 32 | 1 | 1114.34 | 0 | 637.36 | 190.8 | 28 | 47.5 |
|  | 443.7 | 32 | 1.5 | 1114.34 | 0 | 637.36 | 190.8 | 28 | 51 |
|  | 443.7 | 32 | 2 | 1114.34 | 0 | 637.36 | 190.8 | 28 | 41.75 |
| 5 | 435.6 | 30 | 0 | 1033 | 9.9 | 689 | 183 | 28 | 72 |
|  | 435.6 | 30 | 0.6 | 1033 | 9.9 | 689 | 183 | 28 | 74 |
|  | 435.6 | 30 | 1.2 | 1033 | 9.9 | 689 | 183 | 28 | 73.5 |
|  | 435.6 | 30 | 1.8 | 1033 | 9.9 | 689 | 183 | 28 | 73.1 |
|  | 435.6 | 30 | 2.4 | 1033 | 9.9 | 689 | 183 | 28 | 73 |
|  | 435.6 | 30 | 0 | 1033 | 9.9 | 689 | 183 | 90 | 73 |
|  | 435.6 | 30 | 0.6 | 1033 | 9.9 | 689 | 183 | 90 | 75.8 |
|  | 435.6 | 30 | 1.2 | 1033 | 9.9 | 689 | 183 | 90 | 74.2 |
|  | 435.6 | 30 | 1.8 | 1033 | 9.9 | 689 | 183 | 90 | 74 |
|  | 435.6 | 30 | 2.4 | 1033 | 9.9 | 689 | 183 | 90 | 73.8 |
|  | 435.6 | 30 | 0 | 1033 | 9.9 | 689 | 183 | 180 | 73.5 |
|  | 435.6 | 30 | 0.6 | 1033 | 9.9 | 689 | 183 | 180 | 78.6 |
|  | 435.6 | 30 | 1.2 | 1033 | 9.9 | 689 | 183 | 180 | 74.5 |
|  | 435.6 | 30 | 1.8 | 1033 | 9.9 | 689 | 183 | 180 | 75.1 |
|  | 435.6 | 30 | 2.4 | 1033 | 9.9 | 689 | 183 | 180 | 75 |
|  | 435.6 | 30 | 0 | 1033 | 9.9 | 689 | 183 | 365 | 76.5 |
|  | 435.6 | 30 | 0.6 | 1033 | 9.9 | 689 | 183 | 365 | 82.2 |
|  | 435.6 | 30 | 1.2 | 1033 | 9.9 | 689 | 183 | 365 | 80 |
|  | 435.6 | 30 | 1.8 | 1033 | 9.9 | 689 | 183 | 365 | 79 |
|  | 435.6 | 30 | 2.4 | 1033 | 9.9 | 689 | 183 | 365 | 78.5 |
|  | 435.6 | 30 | 0 | 1033 | 9.9 | 689 | 183 | 546 | 77.2 |
|  | 435.6 | 30 | 0.6 | 1033 | 9.9 | 689 | 183 | 546 | 86.3 |
|  | 435.6 | 30 | 1.2 | 1033 | 9.9 | 689 | 183 | 546 | 81.5 |
|  | 435.6 | 30 | 1.8 | 1033 | 9.9 | 689 | 183 | 546 | 82.1 |
|  | 435.6 | 30 | 2.4 | 1033 | 9.9 | 689 | 183 | 546 | 79 |
| 6 | 320 | 10 | 1 | 1171.2 | 0 | 710.4 | 176 | 3 | 16.8 |
|  | 320 | 10 | 2 | 1171.2 | 0 | 710.4 | 176 | 3 | 17.3 |
|  | 320 | 10 | 3 | 1171.2 | 0 | 710.4 | 176 | 3 | 18.9 |
|  | 320 | 10 | 4 | 1171.2 | 0 | 710.4 | 176 | 3 | 21 |
|  | 320 | 10 | 5 | 1171.2 | 0 | 710.4 | 176 | 3 | 21.4 |
|  | 320 | 10 | 6 | 1171.2 | 0 | 710.4 | 176 | 3 | 20.9 |
|  | 320 | 10 | 1 | 1171.2 | 0 | 710.4 | 176 | 7 | 22.2 |
|  | 320 | 10 | 2 | 1171.2 | 0 | 710.4 | 176 | 7 | 23 |
|  | 320 | 10 | 3 | 1171.2 | 0 | 710.4 | 176 | 7 | 24.8 |
|  | 320 | 10 | 4 | 1171.2 | 0 | 710.4 | 176 | 7 | 26.3 |
|  | 320 | 10 | 5 | 1171.2 | 0 | 710.4 | 176 | 7 | 26.5 |
|  | 320 | 10 | 6 | 1171.2 | 0 | 710.4 | 176 | 7 | 26 |
|  | 320 | 10 | 1 | 1171.2 | 0 | 710.4 | 176 | 28 | 31.4 |
|  | 320 | 10 | 2 | 1171.2 | 0 | 710.4 | 176 | 28 | 32.7 |
|  | 320 | 10 | 3 | 1171.2 | 0 | 710.4 | 176 | 28 | 34.6 |
|  | 320 | 10 | 4 | 1171.2 | 0 | 710.4 | 176 | 28 | 37 |
|  | 320 | 10 | 5 | 1171.2 | 0 | 710.4 | 176 | 28 | 39.5 |
|  | 320 | 10 | 6 | 1171.2 | 0 | 710.4 | 176 | 28 | 37.5 |
|  | 320 | 20 | 1 | 1171.2 | 0 | 710.4 | 176 | 3 | 19.86 |
|  | 320 | 20 | 2 | 1171.2 | 0 | 710.4 | 176 | 3 | 20.53 |
|  | 320 | 20 | 3 | 1171.2 | 0 | 710.4 | 176 | 3 | 21.76 |
|  | 320 | 20 | 4 | 1171.2 | 0 | 710.4 | 176 | 3 | 18.23 |
|  | 320 | 20 | 5 | 1171.2 | 0 | 710.4 | 176 | 3 | 17.6 |
|  | 320 | 20 | 1 | 1171.2 | 0 | 710.4 | 176 | 7 | 25.7 |
|  | 320 | 20 | 2 | 1171.2 | 0 | 710.4 | 176 | 7 | 26.66 |
|  | 320 | 20 | 3 | 1171.2 | 0 | 710.4 | 176 | 7 | 27.4 |
|  | 320 | 20 | 4 | 1171.2 | 0 | 710.4 | 176 | 7 | 21.9 |
|  | 320 | 20 | 5 | 1171.2 | 0 | 710.4 | 176 | 7 | 20.7 |
|  | 320 | 20 | 1 | 1171.2 | 0 | 710.4 | 176 | 28 | 32.5 |
|  | 320 | 20 | 2 | 1171.2 | 0 | 710.4 | 176 | 28 | 33.73 |
|  | 320 | 20 | 3 | 1171.2 | 0 | 710.4 | 176 | 28 | 34.3 |
|  | 320 | 20 | 4 | 1171.2 | 0 | 710.4 | 176 | 28 | 29.2 |
|  | 320 | 20 | 5 | 1171.2 | 0 | 710.4 | 176 | 28 | 25.8 |
|  | 320 | 50 | 1 | 1171.2 | 0 | 710.4 | 176 | 3 | 17.87 |
|  | 320 | 50 | 2 | 1171.2 | 0 | 710.4 | 176 | 3 | 21.3 |
|  | 320 | 50 | 3 | 1171.2 | 0 | 710.4 | 176 | 3 | 23.9 |
|  | 320 | 50 | 4 | 1171.2 | 0 | 710.4 | 176 | 3 | 15.36 |
|  | 320 | 50 | 5 | 1171.2 | 0 | 710.4 | 176 | 3 | 14.9 |
|  | 320 | 50 | 1 | 1171.2 | 0 | 710.4 | 176 | 7 | 23.7 |
|  | 320 | 50 | 2 | 1171.2 | 0 | 710.4 | 176 | 7 | 27.03 |
|  | 320 | 50 | 3 | 1171.2 | 0 | 710.4 | 176 | 7 | 34.5 |
|  | 320 | 50 | 4 | 1171.2 | 0 | 710.4 | 176 | 7 | 23.03 |
|  | 320 | 50 | 5 | 1171.2 | 0 | 710.4 | 176 | 7 | 21.9 |
|  | 320 | 50 | 1 | 1171.2 | 0 | 710.4 | 176 | 28 | 30.1 |
|  | 320 | 50 | 2 | 1171.2 | 0 | 710.4 | 176 | 28 | 36.97 |
|  | 320 | 50 | 3 | 1171.2 | 0 | 710.4 | 176 | 28 | 43.8 |
|  | 320 | 50 | 4 | 1171.2 | 0 | 710.4 | 176 | 28 | 32.03 |
|  | 320 | 50 | 5 | 1171.2 | 0 | 710.4 | 176 | 28 | 28.41 |
|  | 320 | 60 | 1 | 1171.2 | 0 | 710.4 | 176 | 3 | 16.9 |
|  | 320 | 60 | 2 | 1171.2 | 0 | 710.4 | 176 | 3 | 17.4 |
|  | 320 | 60 | 3 | 1171.2 | 0 | 710.4 | 176 | 3 | 17.63 |
|  | 320 | 60 | 4 | 1171.2 | 0 | 710.4 | 176 | 3 | 18.7 |
|  | 320 | 60 | 5 | 1171.2 | 0 | 710.4 | 176 | 3 | 15.61 |
|  | 320 | 60 | 1 | 1171.2 | 0 | 710.4 | 176 | 7 | 23.1 |
|  | 320 | 60 | 2 | 1171.2 | 0 | 710.4 | 176 | 7 | 26.1 |
|  | 320 | 60 | 3 | 1171.2 | 0 | 710.4 | 176 | 7 | 27.9 |
|  | 320 | 60 | 4 | 1171.2 | 0 | 710.4 | 176 | 7 | 29.5 |
|  | 320 | 60 | 5 | 1171.2 | 0 | 710.4 | 176 | 7 | 24 |
|  | 320 | 60 | 1 | 1171.2 | 0 | 710.4 | 176 | 28 | 31.2 |
|  | 320 | 60 | 2 | 1171.2 | 0 | 710.4 | 176 | 28 | 33.1 |
|  | 320 | 60 | 3 | 1171.2 | 0 | 710.4 | 176 | 28 | 34.9 |
|  | 320 | 60 | 4 | 1171.2 | 0 | 710.4 | 176 | 28 | 37.1 |
|  | 320 | 60 | 5 | 1171.2 | 0 | 710.4 | 176 | 28 | 32.5 |
|  | 320 | 30 | 1 | 1171.2 | 0 | 710.4 | 176 | 3 | 20.61 |
|  | 320 | 30 | 2 | 1171.2 | 0 | 710.4 | 176 | 3 | 23.45 |
|  | 320 | 30 | 3 | 1171.2 | 0 | 710.4 | 176 | 3 | 25.12 |
|  | 320 | 30 | 4 | 1171.2 | 0 | 710.4 | 176 | 3 | 21.01 |
|  | 320 | 30 | 5 | 1171.2 | 0 | 710.4 | 176 | 3 | 16.3 |
|  | 320 | 30 | 1 | 1171.2 | 0 | 710.4 | 176 | 7 | 22.89 |
|  | 320 | 30 | 2 | 1171.2 | 0 | 710.4 | 176 | 7 | 27.33 |
|  | 320 | 30 | 3 | 1171.2 | 0 | 710.4 | 176 | 7 | 30.2 |
|  | 320 | 30 | 4 | 1171.2 | 0 | 710.4 | 176 | 7 | 27.78 |
|  | 320 | 30 | 5 | 1171.2 | 0 | 710.4 | 176 | 7 | 25.81 |
|  | 320 | 30 | 1 | 1171.2 | 0 | 710.4 | 176 | 28 | 29.9 |
|  | 320 | 30 | 2 | 1171.2 | 0 | 710.4 | 176 | 28 | 32.13 |
|  | 320 | 30 | 3 | 1171.2 | 0 | 710.4 | 176 | 28 | 35.17 |
|  | 320 | 30 | 4 | 1171.2 | 0 | 710.4 | 176 | 28 | 33.98 |
|  | 320 | 30 | 5 | 1171.2 | 0 | 710.4 | 176 | 28 | 29.52 |
|  | 320 | 40 | 1 | 1171.2 | 0 | 710.4 | 176 | 3 | 26.53 |
|  | 320 | 40 | 2 | 1171.2 | 0 | 710.4 | 176 | 3 | 21.57 |
|  | 320 | 40 | 3 | 1171.2 | 0 | 710.4 | 176 | 3 | 19.33 |
|  | 320 | 40 | 4 | 1171.2 | 0 | 710.4 | 176 | 3 | 16.71 |
|  | 320 | 40 | 5 | 1171.2 | 0 | 710.4 | 176 | 3 | 14.6 |
|  | 320 | 40 | 1 | 1171.2 | 0 | 710.4 | 176 | 7 | 28.1 |
|  | 320 | 40 | 2 | 1171.2 | 0 | 710.4 | 176 | 7 | 24.97 |
|  | 320 | 40 | 3 | 1171.2 | 0 | 710.4 | 176 | 7 | 23.37 |
|  | 320 | 40 | 4 | 1171.2 | 0 | 710.4 | 176 | 7 | 19.23 |
|  | 320 | 40 | 5 | 1171.2 | 0 | 710.4 | 176 | 7 | 16.63 |
|  | 320 | 40 | 1 | 1171.2 | 0 | 710.4 | 176 | 28 | 35.63 |
|  | 320 | 40 | 2 | 1171.2 | 0 | 710.4 | 176 | 28 | 33.46 |
|  | 320 | 40 | 3 | 1171.2 | 0 | 710.4 | 176 | 28 | 33.16 |
|  | 320 | 40 | 4 | 1171.2 | 0 | 710.4 | 176 | 28 | 32.01 |
|  | 320 | 40 | 5 | 1171.2 | 0 | 710.4 | 176 | 28 | 27.93 |
|  | 510 | 10 | 1 | 331.5 | 0 | 749.7 | 214.2 | 3 | 16.2 |
|  | 510 | 10 | 2 | 331.5 | 0 | 749.7 | 214.2 | 3 | 16.3 |
|  | 510 | 10 | 3 | 331.5 | 0 | 749.7 | 214.2 | 3 | 16.5 |
|  | 510 | 10 | 4 | 331.5 | 0 | 749.7 | 214.2 | 3 | 17.7 |
|  | 510 | 10 | 5 | 331.5 | 0 | 749.7 | 214.2 | 3 | 15.9 |
|  | 510 | 10 | 1 | 331.5 | 0 | 749.7 | 214.2 | 7 | 20 |
|  | 510 | 10 | 2 | 331.5 | 0 | 749.7 | 214.2 | 7 | 21.3 |
|  | 510 | 10 | 3 | 331.5 | 0 | 749.7 | 214.2 | 7 | 21.9 |
|  | 510 | 10 | 4 | 331.5 | 0 | 749.7 | 214.2 | 7 | 22.7 |
|  | 510 | 10 | 5 | 331.5 | 0 | 749.7 | 214.2 | 7 | 20.7 |
|  | 510 | 10 | 1 | 331.5 | 0 | 749.7 | 214.2 | 28 | 26 |
|  | 510 | 10 | 2 | 331.5 | 0 | 749.7 | 214.2 | 28 | 26.2 |
|  | 510 | 10 | 3 | 331.5 | 0 | 749.7 | 214.2 | 28 | 26.5 |
|  | 510 | 10 | 4 | 331.5 | 0 | 749.7 | 214.2 | 28 | 26.9 |
|  | 510 | 10 | 5 | 331.5 | 0 | 749.7 | 214.2 | 28 | 24.4 |
|  | 510 | 20 | 1 | 331.5 | 0 | 749.7 | 214.2 | 3 | 16.8 |
|  | 510 | 20 | 2 | 331.5 | 0 | 749.7 | 214.2 | 3 | 17 |
|  | 510 | 20 | 3 | 331.5 | 0 | 749.7 | 214.2 | 3 | 18.33 |
|  | 510 | 20 | 4 | 331.5 | 0 | 749.7 | 214.2 | 3 | 17.76 |
|  | 510 | 20 | 5 | 331.5 | 0 | 749.7 | 214.2 | 3 | 17.66 |
|  | 510 | 20 | 1 | 331.5 | 0 | 749.7 | 214.2 | 7 | 18.1 |
|  | 510 | 20 | 2 | 331.5 | 0 | 749.7 | 214.2 | 7 | 19.76 |
|  | 510 | 20 | 3 | 331.5 | 0 | 749.7 | 214.2 | 7 | 22.84 |
|  | 510 | 20 | 4 | 331.5 | 0 | 749.7 | 214.2 | 7 | 21.83 |
|  | 510 | 20 | 5 | 331.5 | 0 | 749.7 | 214.2 | 7 | 19.13 |
|  | 510 | 20 | 1 | 331.5 | 0 | 749.7 | 214.2 | 28 | 21.3 |
|  | 510 | 20 | 2 | 331.5 | 0 | 749.7 | 214.2 | 28 | 23.6 |
|  | 510 | 20 | 3 | 331.5 | 0 | 749.7 | 214.2 | 28 | 27.2 |
|  | 510 | 20 | 4 | 331.5 | 0 | 749.7 | 214.2 | 28 | 25.3 |
|  | 510 | 20 | 5 | 331.5 | 0 | 749.7 | 214.2 | 28 | 23.5 |
|  | 510 | 30 | 1 | 331.5 | 0 | 749.7 | 214.2 | 3 | 13.7 |
|  | 510 | 30 | 2 | 331.5 | 0 | 749.7 | 214.2 | 3 | 15.6 |
|  | 510 | 30 | 3 | 331.5 | 0 | 749.7 | 214.2 | 3 | 16.2 |
|  | 510 | 30 | 4 | 331.5 | 0 | 749.7 | 214.2 | 3 | 12.9 |
|  | 510 | 30 | 5 | 331.5 | 0 | 749.7 | 214.2 | 3 | 11.4 |
|  | 510 | 30 | 1 | 331.5 | 0 | 749.7 | 214.2 | 7 | 18.6 |
|  | 510 | 30 | 2 | 331.5 | 0 | 749.7 | 214.2 | 7 | 19.2 |
|  | 510 | 30 | 3 | 331.5 | 0 | 749.7 | 214.2 | 7 | 20.4 |
|  | 510 | 30 | 4 | 331.5 | 0 | 749.7 | 214.2 | 7 | 18.4 |
|  | 510 | 30 | 5 | 331.5 | 0 | 749.7 | 214.2 | 7 | 18.3 |
|  | 510 | 30 | 1 | 331.5 | 0 | 749.7 | 214.2 | 28 | 26 |
|  | 510 | 30 | 2 | 331.5 | 0 | 749.7 | 214.2 | 28 | 26.3 |
|  | 510 | 30 | 3 | 331.5 | 0 | 749.7 | 214.2 | 28 | 26.95 |
|  | 510 | 30 | 4 | 331.5 | 0 | 749.7 | 214.2 | 28 | 25.91 |
|  | 510 | 30 | 5 | 331.5 | 0 | 749.7 | 214.2 | 28 | 24 |
|  | 510 | 40 | 1 | 331.5 | 0 | 749.7 | 214.2 | 3 | 15.2 |
|  | 510 | 40 | 2 | 331.5 | 0 | 749.7 | 214.2 | 3 | 16.8 |
|  | 510 | 40 | 3 | 331.5 | 0 | 749.7 | 214.2 | 3 | 18.9 |
|  | 510 | 40 | 4 | 331.5 | 0 | 749.7 | 214.2 | 3 | 16.2 |
|  | 510 | 40 | 5 | 331.5 | 0 | 749.7 | 214.2 | 3 | 15.9 |
|  | 510 | 40 | 1 | 331.5 | 0 | 749.7 | 214.2 | 7 | 19.9 |
|  | 510 | 40 | 2 | 331.5 | 0 | 749.7 | 214.2 | 7 | 21.89 |
|  | 510 | 40 | 3 | 331.5 | 0 | 749.7 | 214.2 | 7 | 24.49 |
|  | 510 | 40 | 4 | 331.5 | 0 | 749.7 | 214.2 | 7 | 22.14 |
|  | 510 | 40 | 5 | 331.5 | 0 | 749.7 | 214.2 | 7 | 21.09 |
|  | 510 | 40 | 1 | 331.5 | 0 | 749.7 | 214.2 | 28 | 25.87 |
|  | 510 | 40 | 2 | 331.5 | 0 | 749.7 | 214.2 | 28 | 27.8 |
|  | 510 | 40 | 3 | 331.5 | 0 | 749.7 | 214.2 | 28 | 30.01 |
|  | 510 | 40 | 4 | 331.5 | 0 | 749.7 | 214.2 | 28 | 26.78 |
|  | 510 | 40 | 5 | 331.5 | 0 | 749.7 | 214.2 | 28 | 25.1 |
|  | 510 | 50 | 1 | 331.5 | 0 | 749.7 | 214.2 | 3 | 17 |
|  | 510 | 50 | 2 | 331.5 | 0 | 749.7 | 214.2 | 3 | 17.1 |
|  | 510 | 50 | 3 | 331.5 | 0 | 749.7 | 214.2 | 3 | 17.73 |
|  | 510 | 50 | 4 | 331.5 | 0 | 749.7 | 214.2 | 3 | 16.61 |
|  | 510 | 50 | 5 | 331.5 | 0 | 749.7 | 214.2 | 3 | 16.31 |
|  | 510 | 50 | 1 | 331.5 | 0 | 749.7 | 214.2 | 7 | 22.9 |
|  | 510 | 50 | 2 | 331.5 | 0 | 749.7 | 214.2 | 7 | 22.98 |
|  | 510 | 50 | 3 | 331.5 | 0 | 749.7 | 214.2 | 7 | 23.11 |
|  | 510 | 50 | 4 | 331.5 | 0 | 749.7 | 214.2 | 7 | 21.19 |
|  | 510 | 50 | 5 | 331.5 | 0 | 749.7 | 214.2 | 7 | 20.03 |
|  | 510 | 50 | 1 | 331.5 | 0 | 749.7 | 214.2 | 28 | 25.2 |
|  | 510 | 50 | 2 | 331.5 | 0 | 749.7 | 214.2 | 28 | 26 |
|  | 510 | 50 | 3 | 331.5 | 0 | 749.7 | 214.2 | 28 | 26.4 |
|  | 510 | 50 | 4 | 331.5 | 0 | 749.7 | 214.2 | 28 | 24.9 |
|  | 510 | 50 | 5 | 331.5 | 0 | 749.7 | 214.2 | 28 | 24.85 |
| 7 | 450 | 60 | 0 | 1350 | 0 | 675 | 300 | 7 | 13.65 |
|  | 450 | 60 | 1 | 1350 | 0 | 675 | 300 | 7 | 11.65 |
|  | 450 | 60 | 2 | 1350 | 0 | 675 | 300 | 7 | 11.87 |
|  | 450 | 60 | 3 | 1350 | 0 | 675 | 300 | 7 | 11.75 |
|  | 450 | 60 | 4 | 1350 | 0 | 675 | 300 | 7 | 11.78 |
|  | 450 | 60 | 5 | 1350 | 0 | 675 | 300 | 7 | 15.02 |
|  | 450 | 60 | 0 | 1350 | 0 | 675 | 300 | 28 | 22.73 |
|  | 450 | 60 | 1 | 1350 | 0 | 675 | 300 | 28 | 23.25 |
|  | 450 | 60 | 2 | 1350 | 0 | 675 | 300 | 28 | 23.84 |
|  | 450 | 60 | 3 | 1350 | 0 | 675 | 300 | 28 | 23.44 |
|  | 450 | 60 | 4 | 1350 | 0 | 675 | 300 | 28 | 25.7 |
|  | 450 | 60 | 5 | 1350 | 0 | 675 | 300 | 28 | 26.2 |
| 8 | 375 | 30 | 0 | 1028 | 0 | 701 | 210 | 28 | 43.8 |
|  | 375 | 30 | 0.25 | 1028 | 0 | 701 | 210 | 28 | 45.3 |
|  | 375 | 30 | 0.5 | 1028 | 0 | 701 | 210 | 28 | 47.6 |
|  | 375 | 30 | 0.75 | 1028 | 0 | 701 | 210 | 28 | 50.7 |
|  | 375 | 30 | 1 | 1028 | 0 | 701 | 210 | 28 | 51.6 |
|  | 375 | 30 | 1.25 | 1028 | 0 | 701 | 210 | 28 | 52.8 |
|  | 375 | 30 | 1.5 | 1028 | 0 | 701 | 210 | 28 | 53.6 |
|  | 375 | 30 | 1.75 | 1028 | 0 | 701 | 210 | 28 | 55.1 |
|  | 375 | 30 | 2 | 1028 | 0 | 701 | 210 | 28 | 52.9 |
|  | 375 | 30 | 2.25 | 1028 | 0 | 701 | 210 | 28 | 48.5 |
|  | 375 | 30 | 2.5 | 1028 | 0 | 701 | 210 | 28 | 45.1 |
| 9 | 255 | 50 | 0 | 1075 | 0 | 865 | 180 | 28 | 34 |
|  | 255 | 50 | 1 | 1075 | 0.1 | 865 | 180 | 28 | 36.5 |
|  | 255 | 50 | 2 | 1075 | 0.15 | 865 | 180 | 28 | 38 |
|  | 255 | 50 | 3 | 1075 | 0.25 | 865 | 180 | 28 | 35.5 |
|  | 255 | 50 | 0 | 1075 | 0 | 865 | 180 | 28 | 28 |
|  | 255 | 50 | 1 | 1075 | 0.1 | 865 | 180 | 28 | 30 |
|  | 255 | 50 | 2 | 1075 | 0.15 | 865 | 180 | 28 | 31 |
|  | 255 | 50 | 3 | 1075 | 0.25 | 865 | 180 | 28 | 29 |
| 10 | 432.5 | 0 | 1.5 | 865 | 0 | 865 | 207.6 | 28 | 34.07 |
|  | 432.5 | 25 | 1.5 | 835.3 | 0 | 835.3 | 220.22 | 28 | 33.98 |
|  | 432.5 | 50 | 1.5 | 835.3 | 0 | 835.3 | 220.22 | 28 | 32.18 |
|  | 432.5 | 75 | 1.5 | 835.3 | 0 | 835.3 | 220.22 | 28 | 32.07 |
| 11 | 400 | 0 | 0 | 810 | 0 | 810 | 200 | 28 | 25.5 |
|  | 400 | 50 | 2 | 810 | 0 | 810 | 220 | 28 | 24 |
| 12 | 450 | 50 | 1.5 | 1230 | 3 | 615 | 247.5 | 7 | 21.6 |
|  | 450 | 50 | 1.5 | 1230 | 2.6 | 615 | 247.5 | 7 | 17.7 |
|  | 450 | 50 | 1.5 | 1230 | 2.2 | 615 | 247.5 | 7 | 16.4 |
|  | 450 | 50 | 1.5 | 1230 | 1.8 | 615 | 247.5 | 7 | 11.6 |
|  | 450 | 50 | 1.5 | 1230 | 3 | 615 | 247.5 | 28 | 28.9 |
|  | 450 | 50 | 1.5 | 1230 | 2.6 | 615 | 247.5 | 28 | 21.8 |
|  | 450 | 50 | 1.5 | 1230 | 2.2 | 615 | 247.5 | 28 | 20.5 |
|  | 450 | 50 | 1.5 | 1230 | 1.8 | 615 | 247.5 | 28 | 16.6 |
|  | 450 | 50 | 1.5 | 1230 | 3 | 615 | 247.5 | 90 | 31.9 |
|  | 450 | 50 | 1.5 | 1230 | 2.6 | 615 | 247.5 | 90 | 23.8 |
|  | 450 | 50 | 1.5 | 1230 | 2.2 | 615 | 247.5 | 90 | 21.8 |
|  | 450 | 50 | 1.5 | 1230 | 1.8 | 615 | 247.5 | 90 | 18.4 |
| 13 | 435 | 40 | 0 | 750 | 0 | 900 | 248 | 14 | 20.01 |
|  | 435 | 40 | 0.5 | 750 | 0 | 900 | 248 | 14 | 20.09 |
|  | 435 | 40 | 1 | 750 | 0 | 900 | 248 | 14 | 21.19 |
|  | 435 | 40 | 0.5 | 750 | 0 | 900 | 248 | 14 | 18.49 |
|  | 435 | 40 | 1 | 750 | 0 | 900 | 248 | 14 | 24.16 |
|  | 435 | 40 | 0 | 750 | 0 | 900 | 248 | 28 | 23.26 |
|  | 435 | 40 | 0.5 | 750 | 0 | 900 | 248 | 28 | 26.58 |
|  | 435 | 40 | 1 | 750 | 0 | 900 | 248 | 28 | 26.38 |
|  | 435 | 40 | 0.5 | 750 | 0 | 900 | 248 | 28 | 24.75 |
|  | 435 | 40 | 1 | 750 | 0 | 900 | 248 | 28 | 24.19 |
|  | 435 | 40 | 0 | 750 | 0 | 900 | 248 | 56 | 26.91 |
|  | 435 | 40 | 0.5 | 750 | 0 | 900 | 248 | 56 | 34.15 |
|  | 435 | 40 | 1 | 750 | 0 | 900 | 248 | 56 | 29.79 |
|  | 435 | 40 | 0.5 | 750 | 0 | 900 | 248 | 56 | 24.6 |
|  | 435 | 40 | 1 | 750 | 0 | 900 | 248 | 56 | 28.42 |
|  | 500 | 40 | 0 | 680 | 0 | 885 | 250 | 14 | 26.75 |
|  | 500 | 40 | 0.5 | 680 | 0 | 885 | 250 | 14 | 21.05 |
|  | 500 | 40 | 1 | 680 | 0 | 885 | 250 | 14 | 22.32 |
|  | 500 | 40 | 0.5 | 680 | 0 | 885 | 250 | 14 | 24.11 |
|  | 500 | 40 | 1 | 680 | 0 | 885 | 250 | 14 | 20.82 |
|  | 500 | 40 | 0 | 680 | 0 | 885 | 250 | 28 | 30.97 |
|  | 500 | 40 | 0.5 | 680 | 0 | 885 | 250 | 28 | 28.52 |
|  | 500 | 40 | 1 | 680 | 0 | 885 | 250 | 28 | 30.32 |
|  | 500 | 40 | 0.5 | 680 | 0 | 885 | 250 | 28 | 30.82 |
|  | 500 | 40 | 1 | 680 | 0 | 885 | 250 | 28 | 26.01 |
|  | 500 | 40 | 0 | 680 | 0 | 885 | 250 | 56 | 36.69 |
|  | 500 | 40 | 0.5 | 680 | 0 | 885 | 250 | 56 | 36.15 |
|  | 500 | 40 | 1 | 680 | 0 | 885 | 250 | 56 | 34.36 |
|  | 500 | 40 | 0.5 | 680 | 0 | 885 | 250 | 56 | 31.29 |
|  | 500 | 40 | 1 | 680 | 0 | 885 | 250 | 56 | 32.75 |
| 14 | 425 | 0 | 0 | 970 | 2.7 | 585 | 175 | 28 | 60 |
|  | 425 | 0 | 0.15 | 967 | 2.7 | 584 | 175 | 28 | 61 |
|  | 425 | 0 | 0.3 | 966 | 2.7 | 583 | 175 | 28 | 62 |
|  | 425 | 0 | 0.45 | 965 | 2.7 | 582 | 175 | 28 | 63 |
|  | 425 | 0 | 0.6 | 964 | 2.7 | 581 | 175 | 28 | 62 |
|  | 425 | 0 | 0.75 | 964 | 2.7 | 581 | 175 | 28 | 61.5 |
|  | 425 | 0 | 0 | 970 | 2.7 | 585 | 175 | 56 | 67 |
|  | 425 | 0 | 0.15 | 967 | 2.7 | 584 | 175 | 56 | 69 |
|  | 425 | 0 | 0.3 | 966 | 2.7 | 583 | 175 | 56 | 70 |
|  | 425 | 0 | 0.45 | 965 | 2.7 | 582 | 175 | 56 | 71.5 |
|  | 425 | 0 | 0.6 | 964 | 2.7 | 581 | 175 | 56 | 70.5 |
|  | 425 | 0 | 0.75 | 964 | 2.7 | 581 | 175 | 56 | 60 |
|  | 425 | 0 | 0 | 970 | 2.7 | 585 | 175 | 90 | 75 |
|  | 425 | 0 | 0.15 | 967 | 2.7 | 584 | 175 | 90 | 78 |
|  | 425 | 0 | 0.3 | 966 | 2.7 | 583 | 175 | 90 | 79 |
|  | 425 | 0 | 0.45 | 965 | 2.7 | 582 | 175 | 90 | 79.5 |
|  | 425 | 0 | 0.6 | 964 | 2.7 | 581 | 175 | 90 | 79 |
|  | 425 | 0 | 0.75 | 964 | 2.7 | 581 | 175 | 90 | 77 |
| 15 | 440 | 10 | 4.4 | 1010 | 0 | 698 | 205 | 28 | 40.47 |
|  | 440 | 10 | 4.4 | 1010 | 0 | 698 | 205 | 28 | 45.64 |
|  | 440 | 10 | 4.4 | 1010 | 0 | 698 | 205 | 28 | 42.99 |
|  | 440 | 10 | 4.4 | 1010 | 0 | 698 | 205 | 28 | 50.91 |
| 16 | 550 | 40 | 0 | 1200 | 5.5 | 874 | 160 | 3 | 39 |
|  | 550 | 40 | 0.5 | 1200 | 5.5 | 874 | 160 | 3 | 43 |
|  | 550 | 40 | 1 | 1200 | 5.5 | 874 | 160 | 3 | 44 |
|  | 550 | 40 | 1.5 | 1200 | 5.5 | 874 | 160 | 3 | 37 |
|  | 550 | 40 | 2 | 1200 | 5.5 | 874 | 160 | 3 | 29 |
|  | 550 | 40 | 0 | 1200 | 5.5 | 874 | 160 | 7 | 47 |
|  | 550 | 40 | 0.5 | 1200 | 5.5 | 874 | 160 | 7 | 48 |
|  | 550 | 40 | 1 | 1200 | 5.5 | 874 | 160 | 7 | 49.5 |
|  | 550 | 40 | 1.5 | 1200 | 5.5 | 874 | 160 | 7 | 44 |
|  | 550 | 40 | 2 | 1200 | 5.5 | 874 | 160 | 7 | 34 |
|  | 550 | 40 | 0 | 1200 | 5.5 | 874 | 160 | 28 | 74 |
|  | 550 | 40 | 0.5 | 1200 | 5.5 | 874 | 160 | 28 | 75 |
|  | 550 | 40 | 1 | 1200 | 5.5 | 874 | 160 | 28 | 77 |
|  | 550 | 40 | 1.5 | 1200 | 5.5 | 874 | 160 | 28 | 74 |
|  | 550 | 40 | 2 | 1200 | 5.5 | 874 | 160 | 28 | 68 |
| 17 | 380 | 25 | 0 | 875 | 0 | 750 | 171 | 7 | 18 |
|  | 380 | 25 | 0.25 | 875 | 0 | 750 | 171 | 7 | 18.5 |
|  | 380 | 25 | 0.5 | 875 | 0 | 750 | 171 | 7 | 19 |
|  | 380 | 25 | 0.75 | 875 | 0 | 750 | 171 | 7 | 20.5 |
|  | 380 | 25 | 1 | 875 | 0 | 750 | 171 | 7 | 22 |
|  | 380 | 25 | 1.25 | 875 | 0 | 750 | 171 | 7 | 20 |
|  | 380 | 25 | 1.5 | 875 | 0 | 750 | 171 | 7 | 18.5 |
|  | 380 | 25 | 0 | 875 | 0 | 750 | 171 | 14 | 25 |
|  | 380 | 25 | 0.25 | 875 | 0 | 750 | 171 | 14 | 27 |
|  | 380 | 25 | 0.5 | 875 | 0 | 750 | 171 | 14 | 28 |
|  | 380 | 25 | 0.75 | 875 | 0 | 750 | 171 | 14 | 30 |
|  | 380 | 25 | 1 | 875 | 0 | 750 | 171 | 14 | 31.5 |
|  | 380 | 25 | 1.25 | 875 | 0 | 750 | 171 | 14 | 30 |
|  | 380 | 25 | 1.5 | 875 | 0 | 750 | 171 | 14 | 25 |
|  | 380 | 25 | 0 | 875 | 0 | 750 | 171 | 28 | 39 |
|  | 380 | 25 | 0.25 | 875 | 0 | 750 | 171 | 28 | 40 |
|  | 380 | 25 | 0.5 | 875 | 0 | 750 | 171 | 28 | 41.5 |
|  | 380 | 25 | 0.75 | 875 | 0 | 750 | 171 | 28 | 42.5 |
|  | 380 | 25 | 1 | 875 | 0 | 750 | 171 | 28 | 43 |
|  | 380 | 25 | 1.25 | 875 | 0 | 750 | 171 | 28 | 42 |
|  | 380 | 25 | 1.5 | 875 | 0 | 750 | 171 | 28 | 40 |
|  | 380 | 25 | 0 | 875 | 0 | 750 | 171 | 90 | 41 |
|  | 380 | 25 | 0.25 | 875 | 0 | 750 | 171 | 90 | 42 |
|  | 380 | 25 | 0.5 | 875 | 0 | 750 | 171 | 90 | 43.5 |
|  | 380 | 25 | 0.75 | 875 | 0 | 750 | 171 | 90 | 44 |
|  | 380 | 25 | 1 | 875 | 0 | 750 | 171 | 90 | 45 |
|  | 380 | 25 | 1.25 | 875 | 0 | 750 | 171 | 90 | 42 |
|  | 380 | 25 | 1.5 | 875 | 0 | 750 | 171 | 90 | 41 |
| 18 | 377 | 100 | 0 | 847 | 0 | 917 | 230 | 3 | 22 |
|  | 377 | 100 | 1 | 847 | 0 | 917 | 230 | 3 | 19 |
|  | 377 | 100 | 3 | 847 | 0 | 917 | 230 | 3 | 23 |
|  | 377 | 100 | 5 | 847 | 0 | 917 | 230 | 3 | 18 |
|  | 377 | 100 | 0 | 847 | 0 | 917 | 230 | 7 | 25 |
|  | 377 | 100 | 1 | 847 | 0 | 917 | 230 | 7 | 24 |
|  | 377 | 100 | 3 | 847 | 0 | 917 | 230 | 7 | 26 |
|  | 377 | 100 | 5 | 847 | 0 | 917 | 230 | 7 | 21 |
|  | 377 | 100 | 0 | 847 | 0 | 917 | 230 | 28 | 35 |
|  | 377 | 100 | 1 | 847 | 0 | 917 | 230 | 28 | 36 |
|  | 377 | 100 | 3 | 847 | 0 | 917 | 230 | 28 | 38 |
|  | 377 | 100 | 5 | 847 | 0 | 917 | 230 | 28 | 36 |
| 19 | 394 | 50 | 0 | 1138 | 0 | 687 | 197 | 7 | 14.8 |
|  | 394 | 50 | 4 | 1138 | 0.788 | 687 | 197 | 7 | 13.5 |
|  | 394 | 50 | 5 | 1138 | 1.57 | 687 | 197 | 7 | 12.5 |
|  | 394 | 50 | 6 | 1138 | 2.36 | 687 | 197 | 7 | 12 |
|  | 394 | 50 | 0 | 1138 | 0 | 687 | 197 | 28 | 25.03 |
|  | 394 | 50 | 4 | 1138 | 0.788 | 687 | 197 | 28 | 23.2 |
|  | 394 | 50 | 5 | 1138 | 1.57 | 687 | 197 | 28 | 22.1 |
|  | 394 | 50 | 6 | 1138 | 2.36 | 687 | 197 | 28 | 21.8 |
| 20 | 350 | 200 | 0 | 1400 | 0 | 700 | 175 | 7 | 16.5 |
|  | 350 | 200 | 0.25 | 1400 | 0 | 700 | 175 | 7 | 16 |
|  | 350 | 200 | 0.5 | 1400 | 0 | 700 | 175 | 7 | 12 |
|  | 350 | 200 | 0.75 | 1400 | 0 | 700 | 175 | 7 | 11 |
|  | 350 | 200 | 1 | 1400 | 0 | 700 | 175 | 7 | 10 |
|  | 350 | 200 | 0 | 1400 | 0 | 700 | 175 | 14 | 21.5 |
|  | 350 | 200 | 0.25 | 1400 | 0 | 700 | 175 | 14 | 19.5 |
|  | 350 | 200 | 0.5 | 1400 | 0 | 700 | 175 | 14 | 23.72 |
|  | 350 | 200 | 0.75 | 1400 | 0 | 700 | 175 | 14 | 22.5 |
|  | 350 | 200 | 1 | 1400 | 0 | 700 | 175 | 14 | 20.84 |
|  | 350 | 200 | 0 | 1400 | 0 | 700 | 175 | 21 | 23.93 |
|  | 350 | 200 | 0.25 | 1400 | 0 | 700 | 175 | 21 | 32.34 |
|  | 350 | 200 | 0.5 | 1400 | 0 | 700 | 175 | 21 | 26.35 |
|  | 350 | 200 | 0.75 | 1400 | 0 | 700 | 175 | 21 | 24.5 |
|  | 350 | 200 | 1 | 1400 | 0 | 700 | 175 | 21 | 23.21 |
|  | 350 | 200 | 0 | 1400 | 0 | 700 | 175 | 28 | 25.1 |
|  | 350 | 200 | 0.25 | 1400 | 0 | 700 | 175 | 28 | 33.85 |
|  | 350 | 200 | 0.5 | 1400 | 0 | 700 | 175 | 28 | 27.62 |
|  | 350 | 200 | 0.75 | 1400 | 0 | 700 | 175 | 28 | 25.3 |
|  | 350 | 200 | 1 | 1400 | 0 | 700 | 175 | 28 | 24.35 |
| 21 | 400 | 50 | 0 | 1368 | 1 | 660 | 160 | 3 | 15 |
|  | 400 | 50 | 1 | 1368 | 1 | 660 | 160 | 3 | 13 |
|  | 400 | 50 | 1.5 | 1368 | 1 | 660 | 160 | 3 | 12.5 |
|  | 400 | 50 | 2 | 1368 | 1 | 660 | 160 | 3 | 13.8 |
|  | 400 | 50 | 2.5 | 1368 | 1 | 660 | 160 | 3 | 11.5 |
|  | 400 | 50 | 3 | 1368 | 1 | 660 | 160 | 3 | 11.1 |
|  | 400 | 50 | 0 | 1368 | 1 | 660 | 160 | 7 | 34 |
|  | 400 | 50 | 1 | 1368 | 1 | 660 | 160 | 7 | 28 |
|  | 400 | 50 | 1.5 | 1368 | 1 | 660 | 160 | 7 | 26 |
|  | 400 | 50 | 2 | 1368 | 1 | 660 | 160 | 7 | 31 |
|  | 400 | 50 | 2.5 | 1368 | 1 | 660 | 160 | 7 | 23 |
|  | 400 | 50 | 3 | 1368 | 1 | 660 | 160 | 7 | 21 |
|  | 400 | 50 | 0 | 1368 | 1 | 660 | 160 | 28 | 49 |
|  | 400 | 50 | 1 | 1368 | 1 | 660 | 160 | 28 | 41 |
|  | 400 | 50 | 1.5 | 1368 | 1 | 660 | 160 | 28 | 44 |
|  | 400 | 50 | 2 | 1368 | 1 | 660 | 160 | 28 | 47 |
|  | 400 | 50 | 2.5 | 1368 | 1 | 660 | 160 | 28 | 36 |
|  | 400 | 50 | 3 | 1368 | 1 | 660 | 160 | 28 | 31 |
| 22 | 373 | 30 | 0 | 1120 | 0 | 560 | 187 | 28 | 28.8 |
|  | 373 | 30 | 3.73 | 1120 | 0 | 560 | 187 | 28 | 30.68 |
|  | 373 | 30 | 7.46 | 1120 | 0 | 560 | 187 | 28 | 31.88 |
|  | 373 | 30 | 11.19 | 1120 | 0 | 560 | 187 | 28 | 29.78 |
|  | 373 | 30 | 14.92 | 1120 | 0 | 560 | 187 | 28 | 27 |
|  | 373 | 30 | 0 | 1120 | 0 | 560 | 187 | 28 | 29 |
|  | 373 | 30 | 3.73 | 1120 | 0 | 560 | 187 | 28 | 30 |
|  | 373 | 30 | 7.46 | 1120 | 0 | 560 | 187 | 28 | 32.36 |
|  | 373 | 30 | 11.19 | 1120 | 0 | 560 | 187 | 28 | 29.62 |
|  | 373 | 30 | 14.92 | 1120 | 0 | 560 | 187 | 28 | 26.18 |
|  | 373 | 30 | 0 | 1120 | 0 | 560 | 187 | 28 | 29 |
|  | 373 | 30 | 3.73 | 1120 | 0 | 560 | 187 | 28 | 30.2 |
|  | 373 | 30 | 7.46 | 1120 | 0 | 560 | 187 | 28 | 32 |
|  | 373 | 30 | 11.19 | 1120 | 0 | 560 | 187 | 28 | 29.8 |
|  | 373 | 30 | 14.92 | 1120 | 0 | 560 | 187 | 28 | 26.5 |
| 23 | 493 | 17 | 0 | 0 | 0 | 1052 | 295 | 3 | 41 |
|  | 493 | 17 | 12.8 | 0 | 0 | 1052 | 294 | 3 | 30 |
|  | 493 | 17 | 32 | 0 | 0 | 1052 | 293 | 3 | 27 |
|  | 493 | 17 | 51.2 | 0 | 0 | 1052 | 292 | 3 | 22 |
|  | 493 | 17 | 0 | 0 | 0 | 1052 | 295 | 7 | 52 |
|  | 493 | 17 | 12.8 | 0 | 0 | 1052 | 294 | 7 | 39 |
|  | 493 | 17 | 32 | 0 | 0 | 1052 | 293 | 7 | 37 |
|  | 493 | 17 | 51.2 | 0 | 0 | 1052 | 292 | 7 | 26 |
|  | 493 | 17 | 0 | 0 | 0 | 1052 | 295 | 14 | 59 |
|  | 493 | 17 | 12.8 | 0 | 0 | 1052 | 294 | 14 | 41 |
|  | 493 | 17 | 32 | 0 | 0 | 1052 | 293 | 14 | 40 |
|  | 493 | 17 | 51.2 | 0 | 0 | 1052 | 292 | 14 | 29 |
|  | 493 | 17 | 0 | 0 | 0 | 1052 | 295 | 28 | 65 |
|  | 493 | 17 | 12.8 | 0 | 0 | 1052 | 294 | 28 | 48 |
|  | 493 | 17 | 32 | 0 | 0 | 1052 | 293 | 28 | 43 |
|  | 493 | 17 | 51.2 | 0 | 0 | 1052 | 292 | 28 | 33 |
|  | 493 | 17 | 0 | 0 | 0 | 1052 | 295 | 56 | 69 |
|  | 493 | 17 | 12.8 | 0 | 0 | 1052 | 294 | 56 | 52 |
|  | 493 | 17 | 32 | 0 | 0 | 1052 | 293 | 56 | 46 |
|  | 493 | 17 | 51.2 | 0 | 0 | 1052 | 292 | 56 | 33 |
| 24 | 373 | 30 | 0 | 1120 | 0 | 560 | 180 | 28 | 29 |
|  | 354.35 | 35 | 1 | 1120 | 0 | 560 | 180 | 28 | 31 |
|  | 354.35 | 35 | 2 | 1120 | 0 | 560 | 180 | 28 | 32 |
|  | 354.35 | 35 | 3 | 1120 | 0 | 560 | 180 | 28 | 30 |
|  | 354.35 | 35 | 4 | 1120 | 0 | 560 | 180 | 28 | 28 |
|  | 335.7 | 35 | 1 | 1120 | 0 | 560 | 180 | 28 | 32 |
|  | 335.7 | 35 | 2 | 1120 | 0 | 560 | 180 | 28 | 33 |
|  | 335.7 | 35 | 3 | 1120 | 0 | 560 | 180 | 28 | 30 |
|  | 335.7 | 35 | 4 | 1120 | 0 | 560 | 180 | 28 | 28 |
|  | 317.05 | 35 | 1 | 1120 | 0 | 560 | 180 | 28 | 30 |
|  | 317.05 | 35 | 2 | 1120 | 0 | 560 | 180 | 28 | 31.5 |
|  | 317.05 | 35 | 3 | 1120 | 0 | 560 | 180 | 28 | 29 |
|  | 317.05 | 35 | 4 | 1120 | 0 | 560 | 180 | 28 | 27 |
|  | 298.4 | 35 | 1 | 1120 | 0 | 560 | 180 | 28 | 29 |
|  | 298.4 | 35 | 2 | 1120 | 0 | 560 | 180 | 28 | 30 |
|  | 298.4 | 35 | 3 | 1120 | 0 | 560 | 180 | 28 | 28 |
|  | 298.4 | 35 | 4 | 1120 | 0 | 560 | 180 | 28 | 26 |
| 25 | 320 | 0 | 0 | 1140 | 0 | 869 | 172.8 | 28 | 29.2 |
|  | 320 | 8.6 | 24.26 | 1140 | 0 | 869 | 172.8 | 28 | 31 |
|  | 320 | 12 | 24.26 | 1140 | 0 | 869 | 172.8 | 28 | 29.7 |
|  | 320 | 12 | 34.55 | 1140 | 0 | 869 | 172.8 | 28 | 29.3 |
|  | 320 | 4 | 11.51 | 1140 | 0 | 869 | 172.8 | 28 | 28 |
|  | 320 | 5.8 | 26.49 | 1140 | 0 | 869 | 172.8 | 28 | 30 |
|  | 320 | 12 | 14.97 | 1140 | 0 | 869 | 172.8 | 28 | 32 |
|  | 320 | 4 | 19.81 | 1140 | 0 | 869 | 172.8 | 28 | 25 |
|  | 320 | 8.6 | 34.55 | 1140 | 0 | 869 | 172.8 | 28 | 27.3 |
|  | 320 | 8.6 | 24.26 | 1140 | 0 | 869 | 172.8 | 28 | 30.4 |
|  | 320 | 12 | 14.97 | 1140 | 0 | 869 | 172.8 | 28 | 31 |
|  | 320 | 9.2 | 16.58 | 1140 | 0 | 869 | 172.8 | 28 | 31.5 |
|  | 320 | 8.6 | 24.76 | 1140 | 0 | 869 | 172.8 | 28 | 30.2 |
|  | 320 | 5.2 | 34.55 | 1140 | 0 | 869 | 172.8 | 28 | 25.6 |
|  | 320 | 6.9 | 11.51 | 1140 | 0 | 869 | 172.8 | 28 | 31 |
| 26 | 500 | 20 | 0 | 1060 | 10 | 679 | 175 | 7 | 47 |
|  | 500 | 20 | 0.25 | 1060 | 10 | 679 | 175 | 7 | 36.9 |
|  | 500 | 20 | 0.5 | 1060 | 10 | 679 | 175 | 7 | 37.33 |
|  | 500 | 20 | 0.1 | 1060 | 10 | 679 | 175 | 7 | 40.03 |
|  | 500 | 20 | 1.5 | 1060 | 10 | 679 | 175 | 7 | 39.88 |
|  | 500 | 20 | 2 | 1060 | 10 | 679 | 175 | 7 | 32.19 |
|  | 500 | 20 | 0 | 1060 | 10 | 679 | 175 | 14 | 53 |
|  | 500 | 20 | 0.25 | 1060 | 10 | 679 | 175 | 14 | 39.67 |
|  | 500 | 20 | 0.5 | 1060 | 10 | 679 | 175 | 14 | 40.17 |
|  | 500 | 20 | 0.1 | 1060 | 10 | 679 | 175 | 14 | 44.67 |
|  | 500 | 20 | 1.5 | 1060 | 10 | 679 | 175 | 14 | 43.6 |
|  | 500 | 20 | 2 | 1060 | 10 | 679 | 175 | 14 | 34.77 |
|  | 500 | 20 | 0 | 1060 | 10 | 679 | 175 | 28 | 67.2 |
|  | 500 | 20 | 0.25 | 1060 | 10 | 679 | 175 | 28 | 45.79 |
|  | 500 | 20 | 0.5 | 1060 | 10 | 679 | 175 | 28 | 46.89 |
|  | 500 | 20 | 0.1 | 1060 | 10 | 679 | 175 | 28 | 51.86 |
|  | 500 | 20 | 1.5 | 1060 | 10 | 679 | 175 | 28 | 47.18 |
|  | 500 | 20 | 2 | 1060 | 10 | 679 | 175 | 28 | 41.36 |
| 27 | 320 | 0 | 0 | 935 | 0 | 983 | 205 | 28 | 21.5 |
|  | 320 | 30 | 0.5 | 935 | 0 | 983 | 205 | 28 | 22.47 |
|  | 320 | 30 | 1 | 935 | 0 | 983 | 205 | 28 | 25.39 |
|  | 320 | 30 | 1.5 | 935 | 0 | 983 | 205 | 28 | 26.93 |
|  | 320 | 50 | 0.5 | 935 | 0 | 983 | 205 | 28 | 28.95 |
|  | 320 | 50 | 1 | 935 | 0 | 983 | 205 | 28 | 27.55 |
|  | 320 | 50 | 1.5 | 935 | 0 | 983 | 205 | 28 | 27.1 |
|  | 320 | 40 | 0.5 | 935 | 0 | 983 | 205 | 28 | 21 |
|  | 320 | 40 | 1 | 935 | 0 | 983 | 205 | 28 | 25.5 |
|  | 320 | 40 | 1.5 | 935 | 0 | 983 | 205 | 28 | 28.9 |
| 28 | 407.6 | 50 | 0 | 1260 | 0 | 720 | 191.5 | 7 | 25 |
|  | 316.33 | 50 | 1.74 | 1218 | 1.73 | 694 | 162.8 | 7 | 27 |
|  | 316.33 | 50 | 3.74 | 1218 | 2.08 | 694 | 162.8 | 7 | 28 |
|  | 316.33 | 50 | 5.34 | 1218 | 2.43 | 694 | 162.8 | 7 | 22 |
|  | 316.33 | 50 | 0 | 1218 | 0 | 694 | 162.8 | 28 | 31 |
|  | 316.33 | 50 | 1.74 | 1218 | 1.73 | 694 | 162.8 | 28 | 33 |
|  | 316.33 | 50 | 3.74 | 1218 | 2.08 | 694 | 162.8 | 28 | 35 |
|  | 316.33 | 50 | 5.34 | 1218 | 2.43 | 694 | 162.8 | 28 | 28 |
| 29 | 300 | 35 | 0.25 | 1200 | 0 | 900 | 175 | 7 | 16 |
|  | 300 | 35 | 0.5 | 1200 | 0 | 900 | 175 | 7 | 16.5 |
|  | 300 | 35 | 0.75 | 1200 | 0 | 900 | 175 | 7 | 14 |
|  | 300 | 35 | 1 | 1200 | 0 | 900 | 175 | 7 | 12 |
|  | 300 | 35 | 0.25 | 1200 | 0 | 900 | 175 | 14 | 18 |
|  | 300 | 35 | 0.5 | 1200 | 0 | 900 | 175 | 14 | 19 |
|  | 300 | 35 | 0.75 | 1200 | 0 | 900 | 175 | 14 | 17 |
|  | 300 | 35 | 1 | 1200 | 0 | 900 | 175 | 14 | 15.5 |
|  | 300 | 35 | 0.25 | 1200 | 0 | 900 | 175 | 21 | 22 |
|  | 300 | 35 | 0.5 | 1200 | 0 | 900 | 175 | 21 | 23 |
|  | 300 | 35 | 0.75 | 1200 | 0 | 900 | 175 | 21 | 18 |
|  | 300 | 35 | 1 | 1200 | 0 | 900 | 175 | 21 | 17 |
|  | 300 | 35 | 0.25 | 1200 | 0 | 900 | 175 | 28 | 24 |
|  | 300 | 35 | 0.5 | 1200 | 0 | 900 | 175 | 28 | 25 |
|  | 300 | 35 | 0.75 | 1200 | 0 | 900 | 175 | 28 | 21.5 |
|  | 300 | 35 | 1 | 1200 | 0 | 900 | 175 | 28 | 21 |
|  | 300 | 35 | 0.25 | 1200 | 0 | 900 | 175 | 7 | 17 |
|  | 300 | 35 | 0.5 | 1200 | 0 | 900 | 175 | 7 | 18 |
|  | 300 | 35 | 0.75 | 1200 | 0 | 900 | 175 | 7 | 16 |
|  | 300 | 35 | 1 | 1200 | 0 | 900 | 175 | 7 | 14 |
|  | 300 | 35 | 0.25 | 1200 | 0 | 900 | 175 | 14 | 20 |
|  | 300 | 35 | 0.5 | 1200 | 0 | 900 | 175 | 14 | 23 |
|  | 300 | 35 | 0.75 | 1200 | 0 | 900 | 175 | 14 | 17.5 |
|  | 300 | 35 | 1 | 1200 | 0 | 900 | 175 | 14 | 16 |
|  | 300 | 35 | 0.25 | 1200 | 0 | 900 | 175 | 21 | 23 |
|  | 300 | 35 | 0.5 | 1200 | 0 | 900 | 175 | 21 | 25 |
|  | 300 | 35 | 0.75 | 1200 | 0 | 900 | 175 | 21 | 20 |
|  | 300 | 35 | 1 | 1200 | 0 | 900 | 175 | 21 | 18 |
|  | 300 | 35 | 0.25 | 1200 | 0 | 900 | 175 | 28 | 25 |
|  | 300 | 35 | 0.5 | 1200 | 0 | 900 | 175 | 28 | 27 |
|  | 300 | 35 | 0.75 | 1200 | 0 | 900 | 175 | 28 | 23.5 |
|  | 300 | 35 | 1 | 1200 | 0 | 900 | 175 | 28 | 22.5 |
| 30 | 475 | 50 | 0 | 1079 | 2.38 | 650 | 166.3 | 28 | 54 |
|  | 475 | 50 | 1 | 1079 | 2.61 | 650 | 166.3 | 28 | 52 |
|  | 475 | 50 | 1.5 | 1079 | 2.85 | 650 | 166.3 | 28 | 48 |
|  | 475 | 50 | 2 | 1079 | 2.97 | 650 | 166.3 | 28 | 46 |
|  | 475 | 50 | 0 | 1079 | 2.38 | 650 | 166.3 | 90 | 58 |
|  | 475 | 50 | 1 | 1079 | 2.61 | 650 | 166.3 | 90 | 56 |
|  | 475 | 50 | 1.5 | 1079 | 2.85 | 650 | 166.3 | 90 | 50.5 |
|  | 475 | 50 | 2 | 1079 | 2.97 | 650 | 166.3 | 90 | 48 |
